# A Review of Instability Mechanisms and Control Technologies for Gas Drainage Boreholes in Soft Coal Seams in China

**DOI:** 10.1021/acsomega.6c06764

**Published:** 2026-08-28

**Authors:** Huanchun Liu, Xiangjun Chen, Jianbing Li, Lin Wang, Ziyao Yang, Jianxin Yu

**Affiliations:** † College of Safety Science and Engineering, 12561 Henan Polytechnic University, Jiaozuo 454003, China; ‡ Collaborative Innovation Center of Coal Work Safety and Clean High Efficiency Utilization, Henan Polytechnic University, Jiaozuo 454003, China; § Henan Tiefulai Equipment Manufacturing Co., Ltd., Pingdingshan 467400, China; ∥ Key Laboratory of Intelligent Construction and Safety Operation and Maintenance of Underground Engineering in Henan Province, Henan Polytechnic University, Jiaozuo 454003, China

## Abstract

In China, soft coal seams make up over 50% of all coal seams. The instability and failure of boreholes in soft coal seams are key factors that result in an average gas drainage rate of less than 30% in China, which has become a “bottleneck” for safe and efficient coal mining. Based on 247 articles from the Web of Science Core Collection database (up to April 30, 2025), this review uses a CiteSpace bibliometric analysis to categorize the research into three distinct temporal stages: the exploration stage (2007–2012), the rapid development stage (2013–2019), and the refined research stage (2020–present). Additionally, it conducts an in-depth analysis of five categories comprising nine key influencing factors for borehole stability. In the field of instability prevention and control, the technical processes and their advantages and disadvantages during the borehole construction period and the service period are comprehensively evaluated. Specifically, during the construction period, three types of chemical grouting reinforcement methods and eight types of drilling technologies are covered. During the service period, the review focuses on three types of borehole instability control technologies, including screen pipe physical support, porous material backfilling, and intelligent monitoring. Finally, five critical current bottlenecks are identified, and six development directions are proposed.

## Graphical abstract

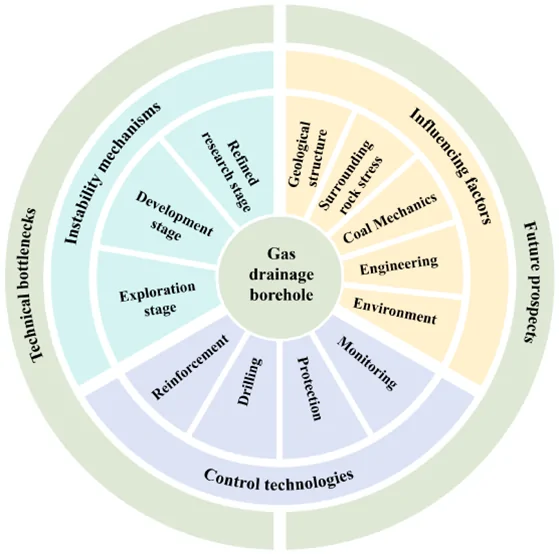

## 1 Introduction

According to statistics from the National Bureau of Statistics of China, coal consumption accounted for 51.4% of the total energy consumption in 2025.^1^ Coal still dominates China’s energy structure and plays an important role in its future economic development.^2^ Coal Mine Methane­(CMM), as an associated resource of coal mining and primarily composed of CH_4_, is a form of clean energy.^3,4^ However, due to its multiple hazard characteristics, the governance of CMM hazards has become an urgent challenge to address. On the one hand, CMM accidents are among the most serious disasters in coal mines.^5^ Approximately 50% of China's coal mines are high gas and outburst mines.^6^ Historically, 20 of the 22 mine accidents with more than 100 fatalities were CMM-related accidents.^7^ On the other hand, over a 100-year time scale, the greenhouse effect of methane is more than 20 times that of CO_2_, and its negative impact on the ozone layer is more than 7 times that of CO_2_.^4,8^ These factors seriously restrict the implementation of China’s “double carbon” strategy. Currently, CMM predrainage is the pivotal technical approach for CMM control,^9^ and holds triple strategic significance. First, it effectively prevents coal and gas outburst. Second, it enhances resource utilization by serving as a clean energy development approach. Third, it contributes to global climate governance through reducing methane emission. These integrated advantages establish CMM drainage as the core technology for synergistically advancing coal mine safety and sustainable development.

Focusing on CMM drainage, scholars have conducted extensive research to improve CMM drainage efficiency through various methods, including hydraulic fracturing,^10,11^ hydraulic slotting,^12,13^ hydraulic punching,^14–17^ liquid CO_2_ fracturing,^18,19^ cumulative blasting,^20^ high-power ultrasonic excitation,^21^ surfactant modification technology,^22^ liquid nitrogen cold soaking^23^ and borehole optimization.^7,24–28^ However, the average CMM drainage rate in Chinese mines is still less than 30%.^29^ Efficient CMM drainage in China is significantly constrained by complex geological conditions, where over 50% of seams are classified as broken soft coal seams.^30^ These seams feature low inherent coal mass strength, well-developed primary fractures and fragmented coal bodies, as well as rheological behavior. Under the influence of in situ stress and mining disturbance, the orifices of CMM drainage boreholes are highly prone to instability, deformation and even collapse.^31–33^ Scholars have pointed out that borehole instability and collapse are the core causes for the decline of gas extraction rate. Studies indicate that the gas concentration of boreholes in soft coal seams drops to less than 5% within 15 days of gas drainage.^34^ As shallow coal resources are progressively depleted,^35^ the depth of coal mining is increasingly moving to deeper strata at a rate of 10–25 m/year.^36^ As burial depth increases, in situ stress escalates and gas pressure rises correspondingly.^37^ Moreover, deep coal seams are typically associated with elevated temperatures and low permeability.^35^ Meanwhile, the roadway structure and mechanical properties of coal-rock masses are also increasingly complex.^38^ The combined effects of the aforementioned conditions progressively weaken the stability of the surrounding rock around boreholes, thereby significantly increasing the risk of borehole instability.

To systematically address CMM drainage borehole instability and enhance extraction efficiency, this study conducts a comprehensive review of literature on the instability of CMM drainage borehole. First, the literature related to CMM drainage borehole instability was visualized using CiteSpace. Based on the Web of Science Core Collection database, a total of 369 articles were retrieved with the topic of (Gas drainage borehole OR gas drainage drilling OR gas drainage borehole OR gas drainage drilling) AND (instability OR failure OR destabilization OR collapse OR stability)’ within the retrieval time frame of before April 30, 2025. After manual screening, 247 related studies were retained.

Based on the statistical analysis of literature retrieval data, the relevant studies have demonstrated a spiral upward trend in recent years (Figure 1a), indicating that the depth of research in this field is continuously increasing. And then focusing on the international cooperation network (Figure 1b), research in this field spans multiple countries. In addition to China, nations like the U.S., Canada, Australia, Germany, and the UK are involved, confirming the global significance of CMM drainage borehole instability. China, with the largest node and darkest color, shows the highest research output and deepest international collaboration. The U.S., Canada, Australia, etc., have more links with China than among themselves, highlighting China’s core role—attributed to its abundant coal resources and complex geological conditions.

**Figure 1:**
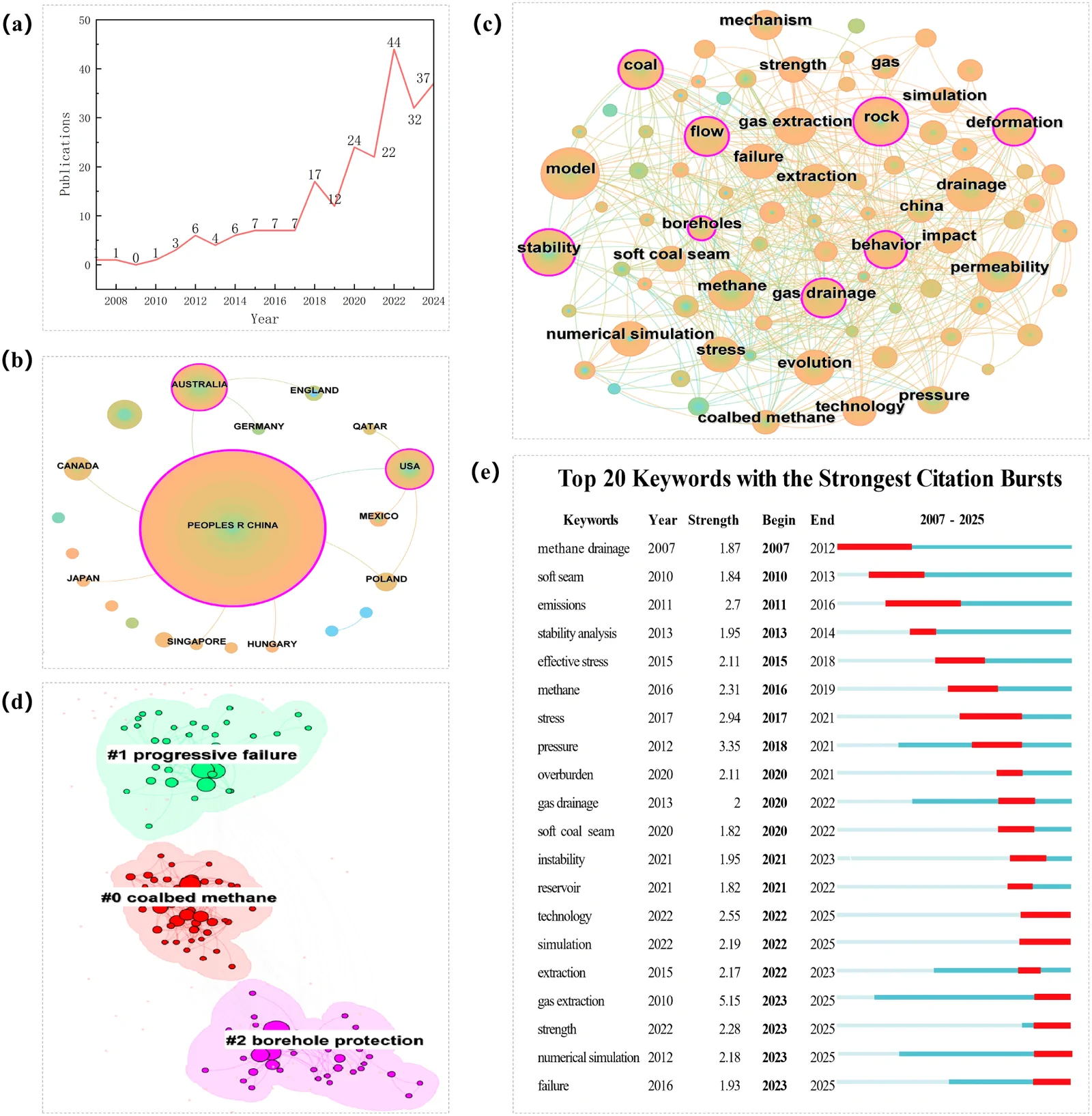
Study on the instability of CMM drainage boreholes based on knowledge graphs bibliometric analysis: (a) Number of publications; (b) International cooperation network map; (c) Keyword co-occurrence map; (d) Keyword cluster map; (e) Keyword burst detection map.

The keyword co-occurrence map (Figure 1c) and the clustering map based on the LLR (Log-Likelihood Ratio) algorithm (Figure 1d) were generated. The modularity (Q = 0.5169 > 0.3) and weighted mean silhouette (S = 0.8066 > 0.7) indicate significant clustering structures.^39^ Among the 11 clusters generated, the top 3 clusters with the highest proportion of literature (accounting for 53% in total) were selected for in-depth analysis. Based on the similarity of themes, the cluster label “*#0 coalbed methane*” was identified as the research focus. The research hotspots are primarily concentrated on two core dimensions. First, the instability mechanisms and influencing factors are explored. High-frequency keywords, including “*stability*”, “*failure*”, “*mechanism*”, “*deformation*”, and “*evolution*”, along with the clustering label “*progressive failure*”, indicate that scholars have conducted systematic research on the instability mechanisms of gas drainage boreholes. Simultaneously, high-frequency keywords including “*coal*”, “*pressure*”, “*stress*”, “*behavior*”, “*permeability*”, and “*impact*” reveal that factors such as uneven stress distribution, coal seam characteristics, and coal seam permeability have become key research objects influencing borehole stability. Second, the research and development of borehole protection technologies are addressed. High-frequency keywords including “*gas drainage*”, “*gas extraction*”, and “*technology*”, along with the clustering label “*borehole protection*”, demonstrate that the development of borehole protection technologies to ensure long-term borehole stability has become a critical research direction for enhancing CMM drainage efficiency and engineering safety. The high frequency of words such as “*model*”, “*numerical simulation*”, and “*evolution*” suggests that numerical simulation and modeling serve as critical technical means and are widely applied in the aforementioned two research dimensions.

Based on the keyword burst detection map (Figure 1e), the research on the instability of CMM drainage boreholes can be divided into three stages. The exploration stage (2007–2012) is characterized by the emergence of keyword bursts such as “*methane drainage*”, “*soft seam*”, “*pressure*”, and “*numerical simulation*”, indicating that the issue of borehole stability in soft coal seams began to attract scholars’ attention during this period. Scholars endeavored to explore the internal mechanism of borehole instability via mechanical theoretical analysis and numerical simulation techniques. The rapid development stage (2013–2019): The prominence of keywords such as “*effective stress*”, “*pressure*”, “*stress*”, and “*stability analysis*” increased significantly. Research on the mechanism of borehole instability based on mechanical theory became mature. Scholars could more accurately construct mechanical models and analyze the influence of different stress on borehole stability. The refinement stage (2020 to present): Keywords such as “*soft coal seam*”, “*instability*”, and “*failure*” saw a sudden surge in frequency, indicating that research in this stage focused on special coal seam conditions like “*soft coal seam*” and core issues like “*instability*” and “*failure*” to explore the internal mechanism of borehole instability in depth. Simultaneously, the increased prominence of the keyword “*technology*” indicates that, in the context of a complex mining environment, technological innovation has become an urgent requirement to address practical problems. The sustained high frequency of keywords such as “*simulation*” and “*numerical simulation*” demonstrates that numerical simulation technology has become the mainstream research approach in this field.

A large number of studies have highlighted the instability issues of CMM drainage boreholes, but a systematic review integrating mechanism analysis and technological innovation remain scare. To fill the research gap, this paper first conducts a bibliometric visual analysis to summarize the failure mechanisms of CMM boreholes. Based on the mechanism analysis, key factors influencing borehole stability were identified. Focusing on the core issues in the borehole construction and service periods, control technologies for borehole instability were classified and summarized. A technical roadmap featuring the “mechanism-technology-bottleneck-prospect” framework was constructed (Figure 2), aiming to provide comprehensive theoretical and technical references for addressing borehole instability.

**Figure 2:**
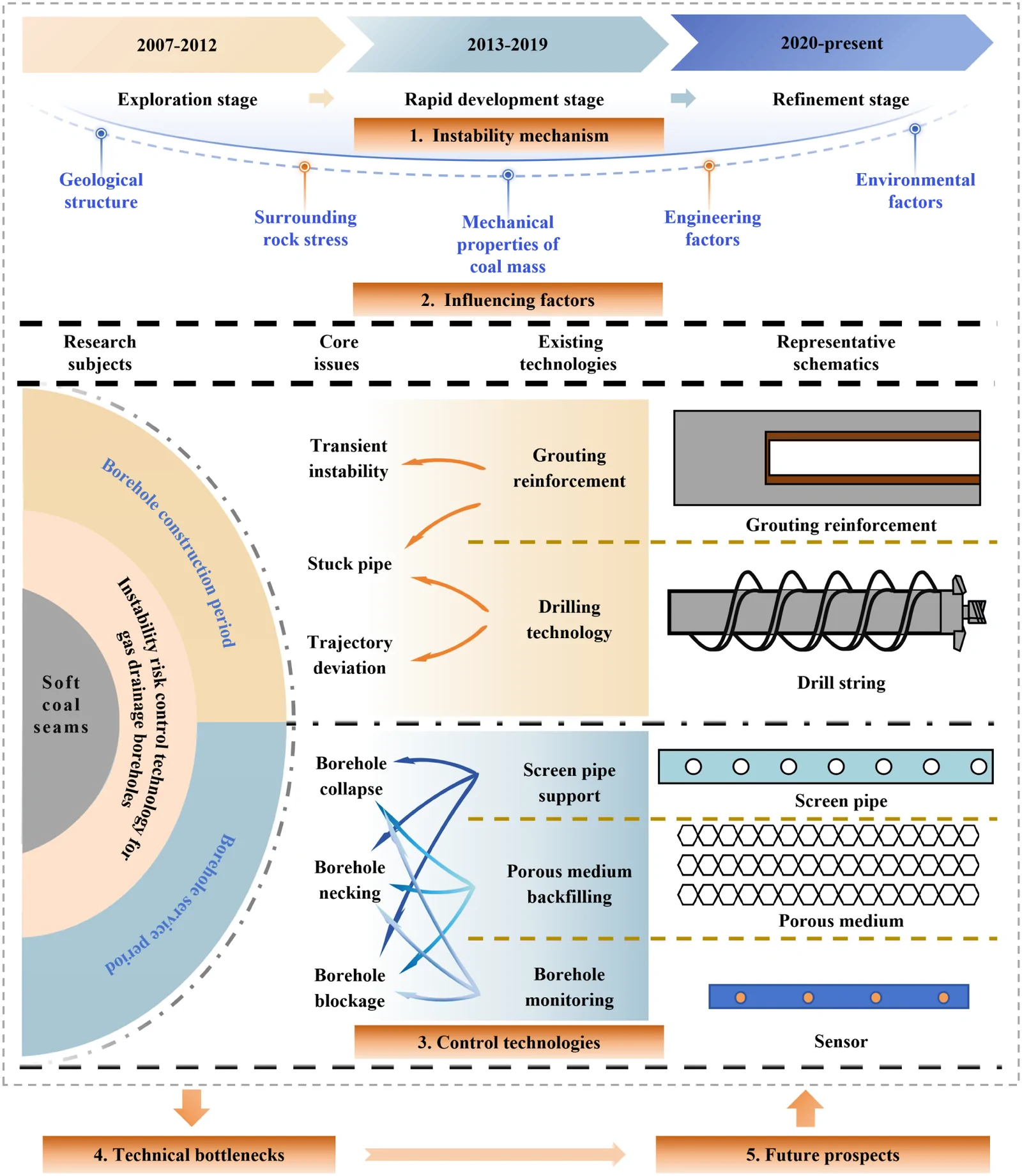
Technical roadmap of this review.

## 2 Instability Mechanisms and Influencing Factors Analysis of CMM Drainage Boreholes

### 2.1 Instability Mechanisms Research of CMM Drainage Boreholes

Based on the bibliometric visual analysis of the literature reviewed in the introduction section, this paper classifies research on gas drainage borehole instability into three development stages, as illustrated in Figure 3.

**Figure 3:**
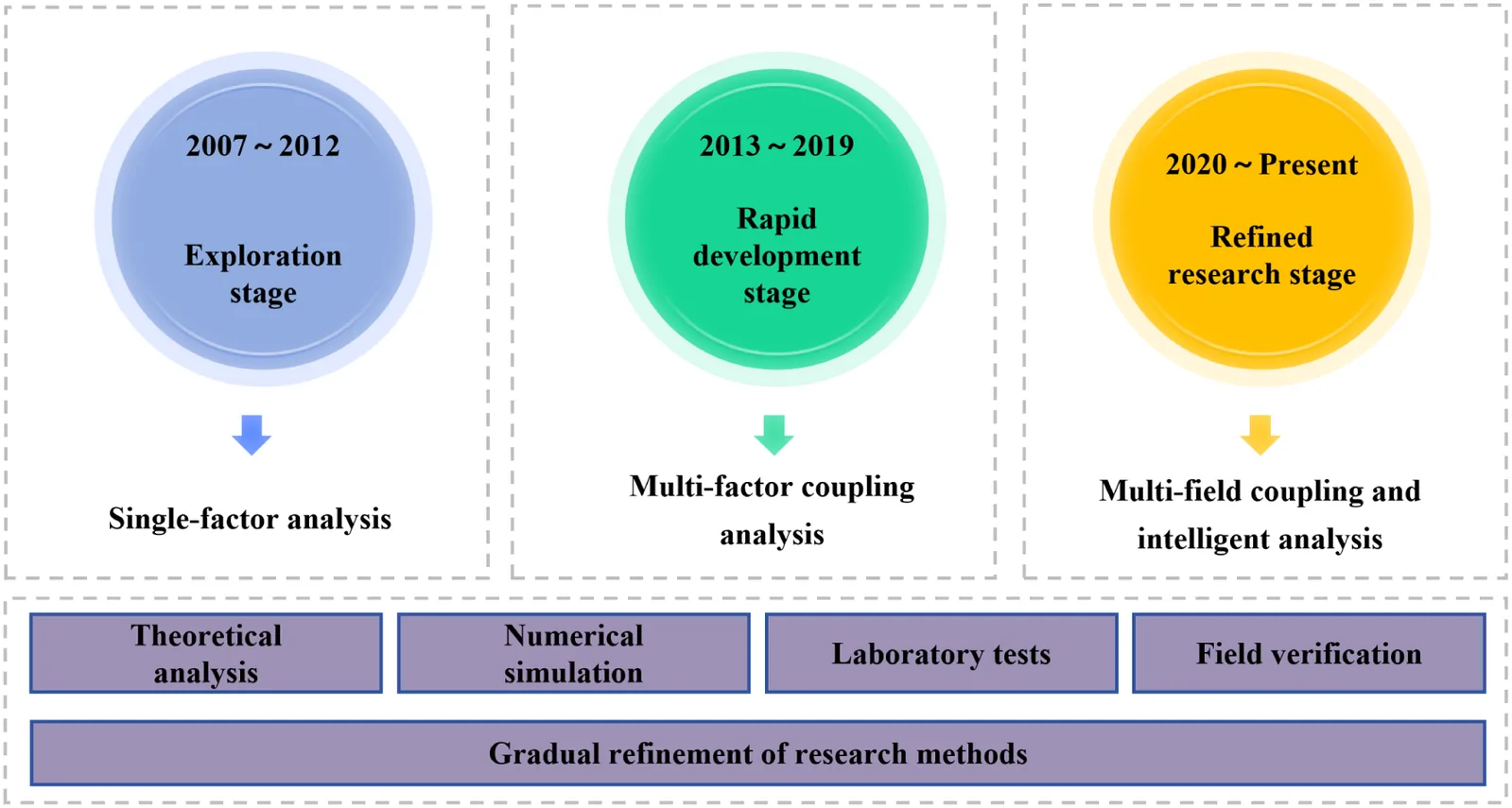
Development stages of borehole instability research.

From 2007 to 2012, research predominantly focused on theoretical analysis, with numerical simulation serving as a supplementary tool. Researchers constructed mechanical models for borehole instability to analyze the failure modes of coal surrounding boreholes and corresponding failure criteria.^40–43^ Wang et al. established a mechanical model of borehole instability and analyzed the failure modes and instability criteria of coal around the borehole wall and bottom. It was found that the stress-relief zone and postpeak stress concentration zone were susceptible to borehole collapse.^42^ Zhai et al. analyzed the deformation mechanism of borehole instability in soft and outburst-prone coal seams in underground coal mines.^41^ It was pointed out that the surrounding rock stress of roadways and the secondary stress of boreholes were the primary causes of the failure and instability of the weak structure zone of borehole wall after fracturing drilling in coal seams. Throughout this period, mechanistic studies on CMM drainage borehole instability remained largely single factor, neglecting the coupling effects of multiple physical fields.

From 2013 to 2019, research on the instability mechanisms of CMM drainage boreholes gradually transitioned from single-factor analysis to multifactor coupled analysis,^33,44–47^ with preliminary explorations conducted on multifield coupling analysis.^48^ During this period, scholars primarily employed theoretical modeling, numerical simulation, and experimental verification to investigate the effects of various factors on borehole stability, including mining activities,^47^ different stress paths,^45,46^ borehole diameter,^44^ and coal-rock bedding.^44^ Relevant borehole instability prevention technologies were also proposed.^47,49^ Liu et al. developed a geomechanical model to characterize the time-dependent evolution of collapse pressure and derived the period of borehole instability.^33^ Zhai et al. analyzed the influence of mining activities on borehole stability and compared the failure behaviors and acoustic emission (AE) evolution between unsupported boreholes and those equipped with internal active support.^47^ Duan et al. simulated layered anisotropic hollow rock samples using the discrete element method.^44^ They investigated the effects of confining pressure, borehole diameter, and anisotropy angle on the mechanical response of the samples and revealed how the borehole instability mechanisms and failure mode changed with the anisotropy angle.^53^ Cheng et al. pointed out that CMM drainage was a multifield coupling process involving the coal-rock damage field, temperature field, and gas seepage field.^48^ They established the damage constitutive equations for coal-rock and deduced the coupling equation of CMM diffusion and seepage in damaged coal-rock. Using numerical simulations, they quantitatively characterized the dynamic evolution of the coal-rock damage field, permeability, and the influence of temperature on gas drainage. Throughout this period, the research scope was broadened, revealing that the instability of CMM drainage boreholes resulted from the coupling effects of multiple factors. Consequently, analyses were conducted by integrating the practical effects of grouting reinforcement and screen pipe support, providing practical guidance for mitigating borehole instability and collapse in soft coal seams.

Since 2020, research on the instability mechanisms of CMM drainage boreholes has entered a stage of refined research, with four prominent characteristics: multifield coupling analysis, investigations on deep and complex geological conditions, research on intelligent technologies, and analysis of dynamic evolution laws of coal-rock fractures. In the realm of multifield coupling analysis, scholars have concentrated on the multifield synergistic effects and established various coupling models, including the damage-stress-seepage coupling model,^24^ the coal-rock continuous damage and binary flow coupling model,^50^ the hydromechanical damage coupling model,^51^ and the thermal-hydraulic-mechanical dual-medium model.^52^ For studies on deep and complex geological environments, scholars have explored borehole instability mechanisms under multiple adverse geological factors, including compound dynamic disasters,^53^ nonuniform stress fields,^54^ high temperature,^52,55^ and water-bearing strata.^52,56–58^ In terms of intelligence, scholars have independently developed borehole monitoring systems using sensing elements such as strain gauges and fiber Bragg gratings to accurately determine the location and degree of borehole instability.^59–62^ Regarding fracture propagation, Pan et al. conducted experimental research on the surface deformation of samples with different drilling spacings during the progressive damage process using a coal-rock deformation and damage test platform based on digital image correlation technology.^63^ Cheng et al. investigated the deformation evolution and fracture propagation mechanisms around CMM drainage boreholes in composite strata under true triaxial loading via internal strain-brick and AE monitoring.^64^ At this stage, scholars have employed a variety of methods to conduct research on the dynamic evolution patterns of borehole instability.

In summary, research on the instability mechanisms of CMM drainage boreholes has evolved from single mechanical model analysis to multifield coupling modeling, and from theoretical analysis to intelligent monitoring. This progression has not only deepened the understanding of borehole instability mechanisms but also effectively promoted the transformation of CMM drainage engineering from passive control to proactive prevention. However, there remain some deficiencies in the study of borehole instability mechanisms. Currently, various theories and numerical models are established by simplifying geological conditions, focusing only on limited influencing factors. Nevertheless, the geological conditions in coal mines are highly complex and uncertain, resulting in a certain deviation between some research results and actual working conditions.

### 2.2 Influencing Factors of CMM Drainage Borehole Instability

Research on the instability mechanisms of CMM drainage boreholes has shown that the stability is influenced by multiple factors, including geological structure, in situ stress, mining-induced stress, gas pressure, mechanical properties of coal mass, support conditions, and environmental factors, as shown in Figure 4.

**Figure 4:**
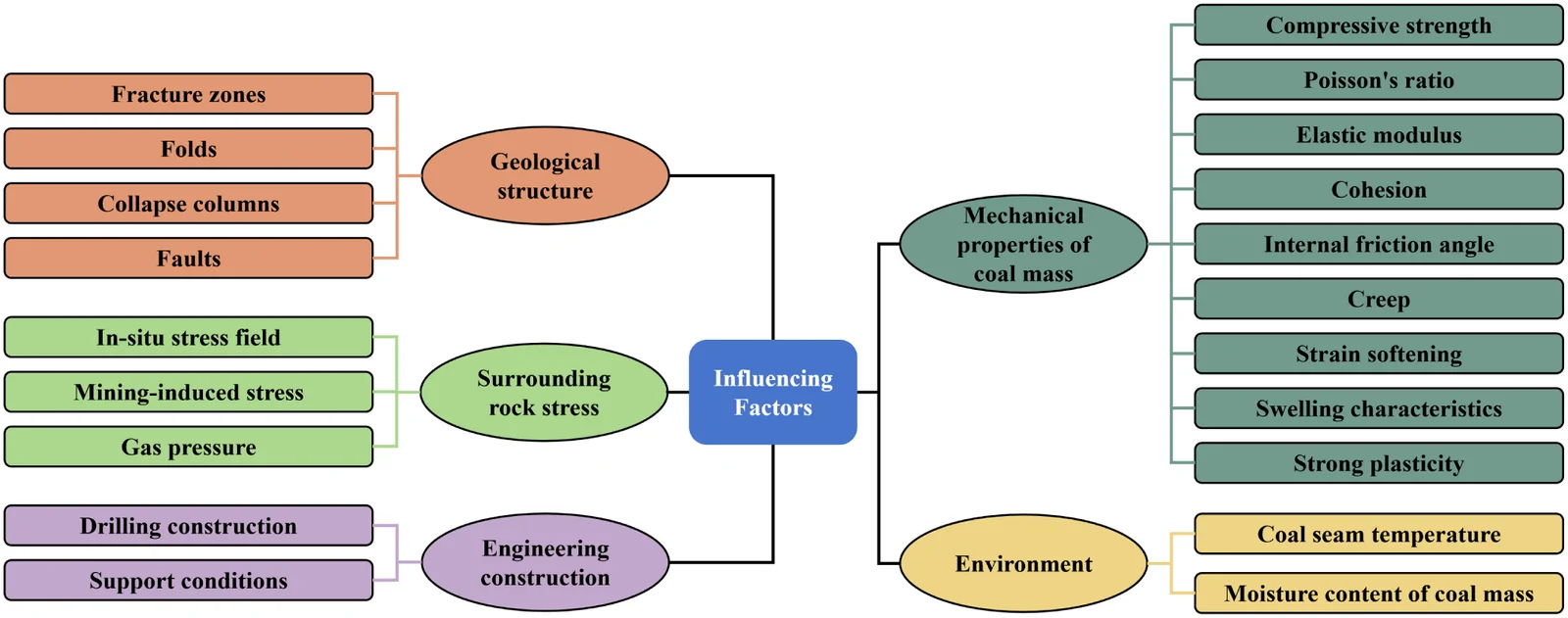
Influencing factors of borehole instability in soft coal seams.

#### 2.2.1 Geological Structure

During long-term geological evolution, diverse geological structures have been formed in coal seams.^65^ The geological structures closely related to the stability of coal seam boreholes in China include fracture zones, folds (synclines and anticlines), collapse columns and faults.^66,67^ The fracture zone is characterized by low strength, proneness to deformation, high water permeability, and weak water resistance. Borehole instability tends to occur when drilling penetrates such fractured zones.^66,67^ During the formation of fold structures, interlayer sliding takes place between strong and weak rock strata, displacing the coal seam from the high-stress zone to the low-stress zone. This process induces local thickness variations, coal mass fragmentation, and the development of fractures along the coal seam.^65^ Meanwhile, in the stress variation zones formed by folds, especially in the tensile stress zones, the coal mass has low strength, which easily causes instability and failure of boreholes.^66,67^ Karst collapse columns, as a special geological structure in the Carboniferous–Permian coalfields of northern China, are widely distributed in 45 mining areas across 20 coalfields.^68^ Under mining disturbance, karst collapse columns can connect the upper and lower aquifers to form unobstructed water-conduction channels. Additionally, in the deep covered areas, they exhibit diverse geometries and are difficult to detect.^69,70^ During the drilling process, if collapse columns are encountered, the filling materials of the collapse column may loosen and migrate under the action of water flow,^70^ affecting the stability of the borehole. Faults are one of the most common geological structures in underground coal mines. When activated by mining activities, they can trigger major disasters such as rockburst, coal and gas outburst, fault-related water inrushes, and mining-induced seismicity.^71^ Faults induce abnormal stress concentrations in tectonic areas, where the coupled effect of dynamic and static loads imposes excessive stress on the coal mass, leading to coal mass fragmentation and displacement.^72^ As the working face advances toward faults, the mining process transitions from stable primary coal bodies to fragmented tectonically disturbed coal mass.^71^ The abrupt lithological contrast across faults forms abnormal soft–hard transition zones, which readily trigger borehole instability and pipe sticking during drilling.^66,67^ In geologically complex areas with soft coal seams, borehole instability, collapse, and blockage are extremely prone to occur across entire or local borehole sections. Such instability typically manifests during borehole construction, but may progressively evolve into complete borehole blockage over time.^66^

#### 2.2.2 Surrounding Rock Stress

Borehole instability and collapse primarily stem from shear failure, which occurs when the stress in the coal surrounding the borehole exceeds the coal’s inherent strength.^42^ Thus, the stress state of the surrounding rock—including in situ stress, mining-induced stress field, and CMM pressure—represents a critical factor influencing the stability of CMM drainage boreholes.

##### 2.2.2.1 In-Situ Stress

In-situ stress consists mainly of overburden stress and tectonic stress. Both burial depth and the lateral pressure coefficient determine the magnitude and distribution of in situ stress. Based on the geostress data, horizontal principal stress rises monotonically with the increase of burial depth,^73^ which is consistent with the field measurement results of deep coal mines reported in previous research.^41^ Through discrete element simulation, Sun et al. verified that the rise of burial depth elevates the overall geostress level, aggravates coal deformation and fracture propagation around boreholes, and markedly reduces borehole stability. The lateral pressure coefficient exerts an obvious regulating effect on instability of boreholes. Zhang et al. found that both the maximum vertical and horizontal displacements of the borehole surrounding rock increase with rising lateral pressure coefficient, accompanied by a slow increase in the maximum vertical stress at the borehole.^74^ Meanwhile, when the lateral pressure coefficient exceeds 1 and horizontal stress dominates the in situ stress field, extrusion damage and fracture propagation of coal-rock around boreholes will be greatly intensified.^75^ Zhang et al. reported that in situ stress, governed by burial depth and lateral pressure coefficient, is the fundamental cause of borehole instability and the primary factor controlling borehole stability.^74^

##### 2.2.2.2 Mining-Induced Stress Analysis

Coal-rock masses are disturbed by three types of mining activities, including roadway excavation, drilling exploration, and coal seam mining.^47^ At the initial stage, the in situ stress equilibrium between the roadway and the rock mass is disrupted by roadway excavation, resulting in a redistribution of the magnitude and direction of stress in the surrounding rock. Along the direction perpendicular to the roadway, the borehole is identified as four distinct zones: the stress reduction zone, postpeak stress increase zone, prepeak stress increase zone, and original rock stress zone.^41,42,47^ Among them, in the stress reduction zone, coal undergoes plastic deformation after reaching peak stress, generating numerous fractures. Elastic potential energy is completely dissipated, resulting in the lowest strength, where slight disturbance may induce instability, deformation, or even collapse. In the postpeak stress increase zone, stress exceeds the strength limit of coal, leading to the formation of numerous microfractures within the coal. The coal is in a softening deformation stage, making borehole walls susceptible to instability.^41^ By contrast, the prepeak stress increase zone has a significantly lower likelihood of instability, while the original rock stress zone exhibits the highest stability.^42^ Subsequently, drilling construction exacerbates the stress perturbation around the coal, building on the stress field modified by prior roadway excavation. Along the borehole diameter direction, from inside to outside, the zones can be classified as the fracture zone, plastic zone, elastic zone, and original stress zone. In the fracture zone, coal exhibits the weakest resistance to external disturbances and is most prone to collapse.^41,47^ Finally, coal seam mining acts as a tertiary disturbance, inducing dynamic adjustments in both stress magnitude and principal stress trajectories ahead of the advancing working face. Along the coal seam direction, three zones can be identified: the stress reduction zone, stress increase zone, and stress stable zone. The stress increase zone is most prone to borehole instability.^47^ Due to the long-term and repeated effects of various mining activities, the coal around the borehole is highly susceptible to plastic deformation and even collapse.

##### 2.2.2.3 Gas Pressure Analysis

Gao et al. found that gas pressure acts as a volumetric force on both the coal matrix skeleton and fractures, inducing a dilatancy effect that leads to matrix expansion.^76^ The higher the gas pressure, the more pronounced the coal dilatancy becomes. Gao et al. reported that increasing gas pressure can degrade the compressive strength and elastic modulus of coal samples, while Poisson’s ratio presents an upward trend.^77^ Additionally, Zhang et al. demonstrated a positive correlation between CMM pressure and borehole stability.^78^ As CMM pressure increases, the maximum vertical displacement, maximum horizontal displacement, and peak vertical stress of the rock mass exhibit a gradual upward trend. Furthermore, Wang et al. revealed that rising CMM pressure promotes coal fracture propagation, enhances crack density, and aggravates fragmentation severity.^79,80^ Notably, Liu et al. observed an approximately linear relationship between the original CMM pressure and maximum collapse pressure, wherein higher collapse pressures prolong the borehole instability risk period.^33^

#### 2.2.3 Mechanical Properties of Coal Mass

Liu et al. pointed out that most coal seams in China formed during the Carboniferous-Permian period.^81^ Consequently, the coal has experienced multiple intense tectonic movements, which have disrupted original fractures within the coal and transformed it into a soft, structurally complex coal mass. Stress concentration in the borehole vicinity readily induces borehole failure, particularly in soft coal seams.^84^ Soft coal seams are characterized by a uniaxial compressive strength of less than 5.0 MPa,^34^ lower cohesion and elastic modulus compared to hard coals, and higher Poisson’s ratio.^82^ They also exhibit low failure resistance, creep characteristics, strain softening, swelling properties,^34^ strong plasticity,^83^ low internal friction angles,^84^ and low permeability.^83,85^ These characteristics result in more intense creep deformation of the surrounding coal. The borehole deformation develops rapidly, and complete borehole collapse and blockage can occur within a short period, leading to failure of the gas drainage channel, as shown in Figure 5.^34^ Furthermore, compared with hard coal, soft coal exhibits larger deformation under a lower peak stress,^86^ which makes the boreholes more prone to instability and failure in a short time.

**Figure 5:**
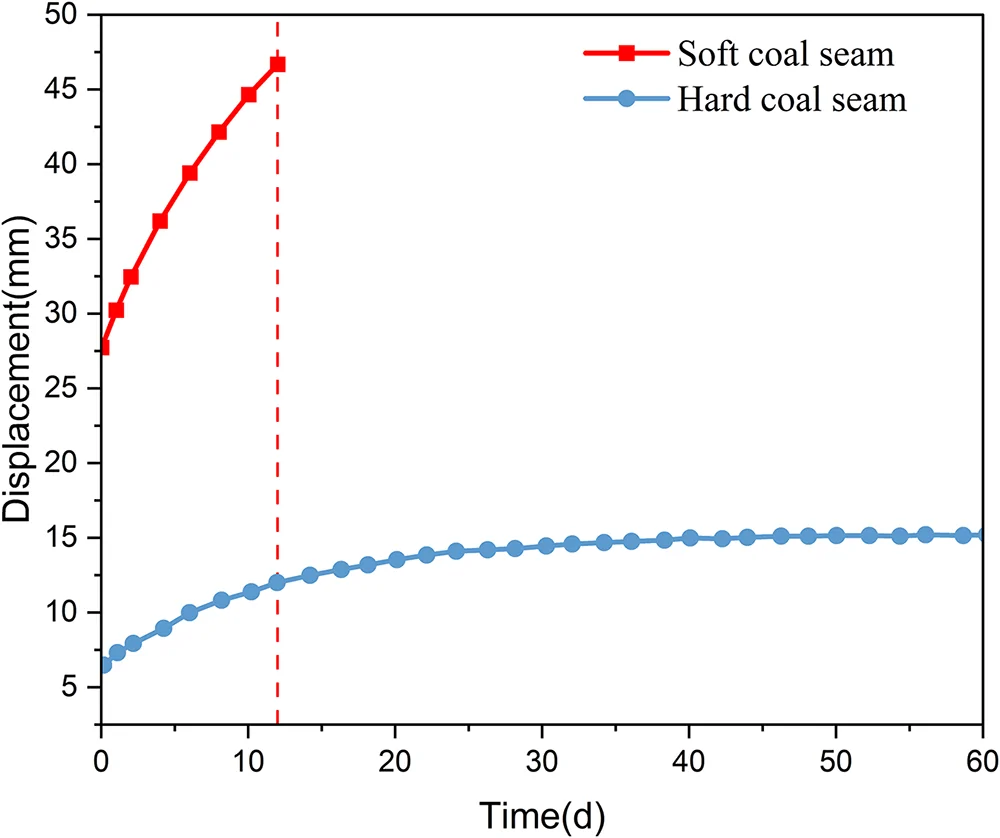
Displacements of borehole wall in soft and hard coal seams at different extraction times. Experimental data are extracted and replotted from ref 34 (Hao, F.; Sun, L.; Zhao, F. Borehole diameter shrinkage rule considering rheological properties and its effect on gas extraction. *PLoS One* **2020**, *15* (9), e0239016), originally published under CC BY 4.0 license. License: https://creativecommons.org/licenses/by/4.0/.

#### 2.2.4 Engineering Factors

##### 2.2.4.1 Borehole Construction

During borehole construction, factors governing the borehole stability can be divided into two categories: drilling disturbance effects and borehole construction parameters.^31,87–91^ The drill pipe is subjected to the combined effects of axial thrust, self-weight, torque, and borehole wall friction during drilling. With increasing borehole depth, rising axial pressure causes the pipe to bend, which exacerbates mechanical disturbance to the borehole wall and may ultimately induce wall instability or borehole failure.^88^ A high rate of penetration intensifies mechanical disturbance on the borehole wall and substantially increases cuttings production. On the one hand, the accumulation of cuttings significantly raises the resistance to transport in the annulus; on the other hand, soft coal undergoes plastic deformation under in situ stress and drilling-induced disturbance, progressively narrowing the annular space between the drill pipe and the borehole wall. These two factors jointly reduce the pneumatic cuttings-transport capacity of the drilling system. Once cuttings production exceeds the system’s maximum transport capacity, accumulation and blockage occur, further disturbing and compressing the surrounding coal mass, aggravating plastic failure of the borehole wall, and ultimately inducing borehole instability.^90^ The borehole diameter also has a notable impact on the strength of the borehole. Wang et al. conducted uniaxial compression tests on sandstone specimens with prefabricated boreholes and found that the elastic energy storage limit of rock specimens decreases while the degree of plastic deformation rises with the increase of borehole diameter. As illustrated in Figure 6, when the borehole diameter increases from 8 mm to 14 mm, the peak elastic energy of rock specimens drops from 23.61 kJ/m^3^ to 10.07 kJ/m^3^. Meanwhile, the modified brittleness index (BIM) calculated based on energy distribution rises from 0.59 to 1.35, which indicates that larger boreholes correspond to more prominent plastic deformation, accompanied by weakened brittleness and intensified ductile damage characteristics. Although the experiments carried out by Wang et al. adopted sandstone samples, the revealed law also provides valuable reference for understanding the progressive failure mechanism of coal mass surrounding gas drainage boreholes in soft coal seams.^87^ Zhao et al. found through underground field monitoring that, in gas extraction drilling holes of the same aperture, the deformation magnitude of the surrounding rock in the shallow section is significantly greater than that in the deep section; this phenomenon is attributed to more severe structural damage to the shallow coal mass and more complete release of ground stress.^92^ Meanwhile, numerous scholars have conducted research on the mechanism of borehole instability induced by drilling fluids. Cao et al. verified via SEM and CT tests that face cleats, butt cleats and bedding fractures are well developed within coal rock.^93^ Liu et al. further validated through mechanical immersion tests with drilling fluids that filtrate intrusion along fractures significantly degrades the mechanical properties of coal rock; after 24 h of immersion, the compressive strength of coal rock drops to only 55%–65% of that of dry specimens.^94^ Additionally, different drilling technologies have a significant impact on wellbore stability. We will elaborate on this in Section 3.1.2.

**Figure 6:**
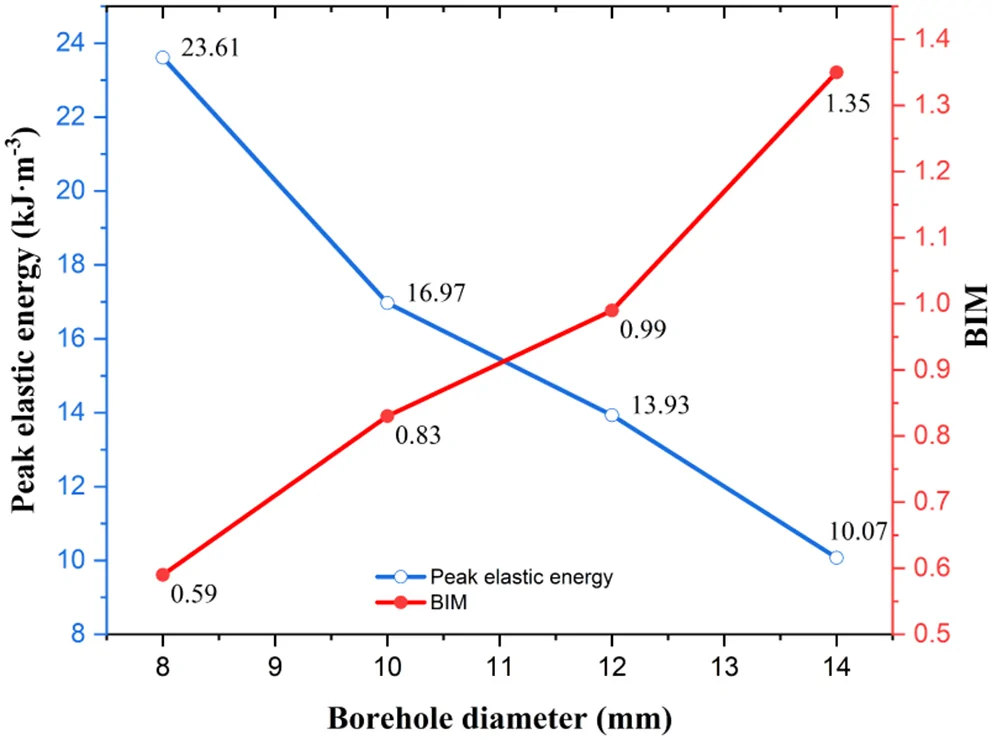
Variation of peak elastic energy and brittleness index modification (BIM) with borehole diameter. Experimental data are extracted and replotted from ref 87 (Wang, Y.; Xu, M.; Kong, D.; Wu, G.; Shang, Y.; Chen, L.; Liu, Y. Study on destabilization failure characteristics and energy evolution law of drilling holes. *Energy Science & Engineering* **2023**, *11*(8), 2818–2830), originally published under CC BY 3.0 license. License: https://creativecommons.org/licenses/by/3.0/.

##### 2.2.4.2 Support Conditions

Scholars have conducted in-depth research on the stability of CMM drainage boreholes under three conditions: with and without screen pipe support, and with borehole reinforcement measures. The results show that without screen pipe support, the maximum vertical and horizontal displacements in the stress concentration zone and surrounding rock of the borehole are significantly larger. Screen pipe support with different materials reduces the scope of the stress concentration zone and the maximum vertical and horizontal displacements to varying degrees.^74^ Although screen pipe support cannot alter the mechanical properties of the borehole surrounding coal, it can stabilize the coal in the fault zone and keep it in situ, reduce vertical displacement and alleviate stress concentration around the borehole, thereby effectively maintaining the integrity of the CMM emission channel. Results show that screen pipe support enhances the borehole’s stability by at least 40%.^47,95^ Additionally, injecting reinforcement materials into the borehole to actively support the borehole wall can enhance the stability of coal mass around the borehole.^50,96^ Boreholes subjected to grouting reinforcement sustain elevated methane concentrations over prolonged periods, demonstrating that the surrounding rock mass has been effectively stabilized and that borehole collapse has been inhibited.^96^ Thus, installing screen pipes or injecting reinforcement materials in CMM drainage boreholes within soft coal seams significantly improves borehole stability and reduces borehole collapse risks.

#### 2.2.5 Environmental Factors

##### 2.2.5.1 Coal Seam Temperature

With the increasing depth of coal mining, the temperature of coal seams gradually rises and their conditions continue to deteriorate, leading to frequent coal-rock dynamic disasters.^48^ Temperature rise can promote gas desorption and reduce adsorption swelling deformation,^97,98^ while the creep strain of coal increases simultaneously.^98^ Wang et al. found that temperature rise increases the proportion of free gas in fractures. The gas pressure counteracts part of the in situ stress, which reduces the effective stress and deteriorates the mechanical properties of coal. When the coal seam temperature rises from 25 to 55 °C, the peak strength and peak strain of coal decrease by 37.1% and 42.4%, respectively, while the elastic modulus exhibits an evolution characteristic of rising first and then falling, as shown in Figure 7. A moderate temperature rise only compacts primary fractures and temporarily improves coal stiffness; sustained high temperature generates abundant micro cracks and accumulates internal damage, weakening the bearing and energy storage capacity of coal, which easily triggers failure of the surrounding rock’s energy storage structure.^6^

**Figure 7:**
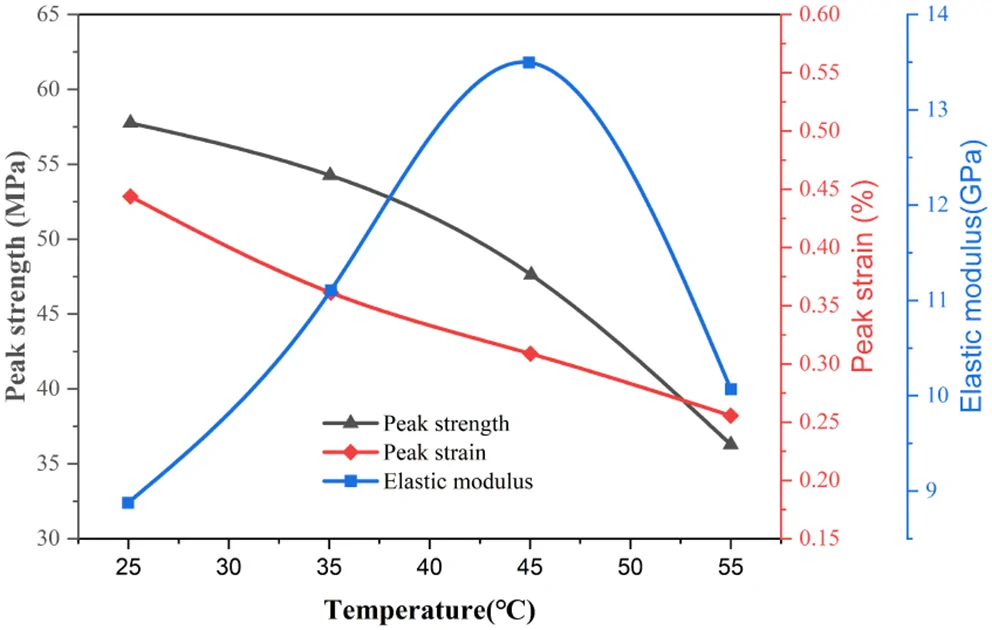
Evolution of mechanical parameters of coal under different temperatures. Experimental data are extracted and replotted from ref 6 (Wang, W.; Yang, W.; Zhang, Z.; Shi, W. Multifactor analysis and dominant factor identification for coal seam gas drainage borehole instability. *Scientific Reports* **2026**, *16* (1), 2575), originally published under CC BY 4.0 license. License: https://creativecommons.org/licenses/by/4.0/.

##### 2.2.5.2 Moisture Content of Coal Mass

The presence of water in coal mines also significantly affects borehole stability to some extent. Guo et al. compared graded loading creep curves of hole-containing specimens with four water contents (0%, 10.4%, 19.3%, 28.4%) (Figure 8) and found that the growth of axial strain over time rises with increasing water content. Water exerts physical erosion and softening effects on porous coal specimens. From a microscopic perspective, water wets the free-surface coal particles, weakens cohesion, degrades the overall mechanical properties of coal, and renders the stressed coal mass around boreholes more susceptible to creep deformation.^56^ Gao et al. verified that moisture content exerts an obvious two-way regulatory effect on the mechanical properties of coal mass. Moderate moisture can enhance coal strength via liquid bridge forces between particles; nevertheless, once the moisture content exceeds the optimal critical value of 9%, excessive water will drastically reduce the uniaxial compressive strength, elastic modulus, cohesion and internal friction angle of coal, and degrade its bearing and shear resistance.^99^ In summary, when the moisture content of coal mass exceeds the critical value, the bearing capacity and shear resistance of coal will decline simultaneously, accompanied by continuous development of creep deformation, which significantly increases the risk of surrounding rock instability.

**Figure 8:**
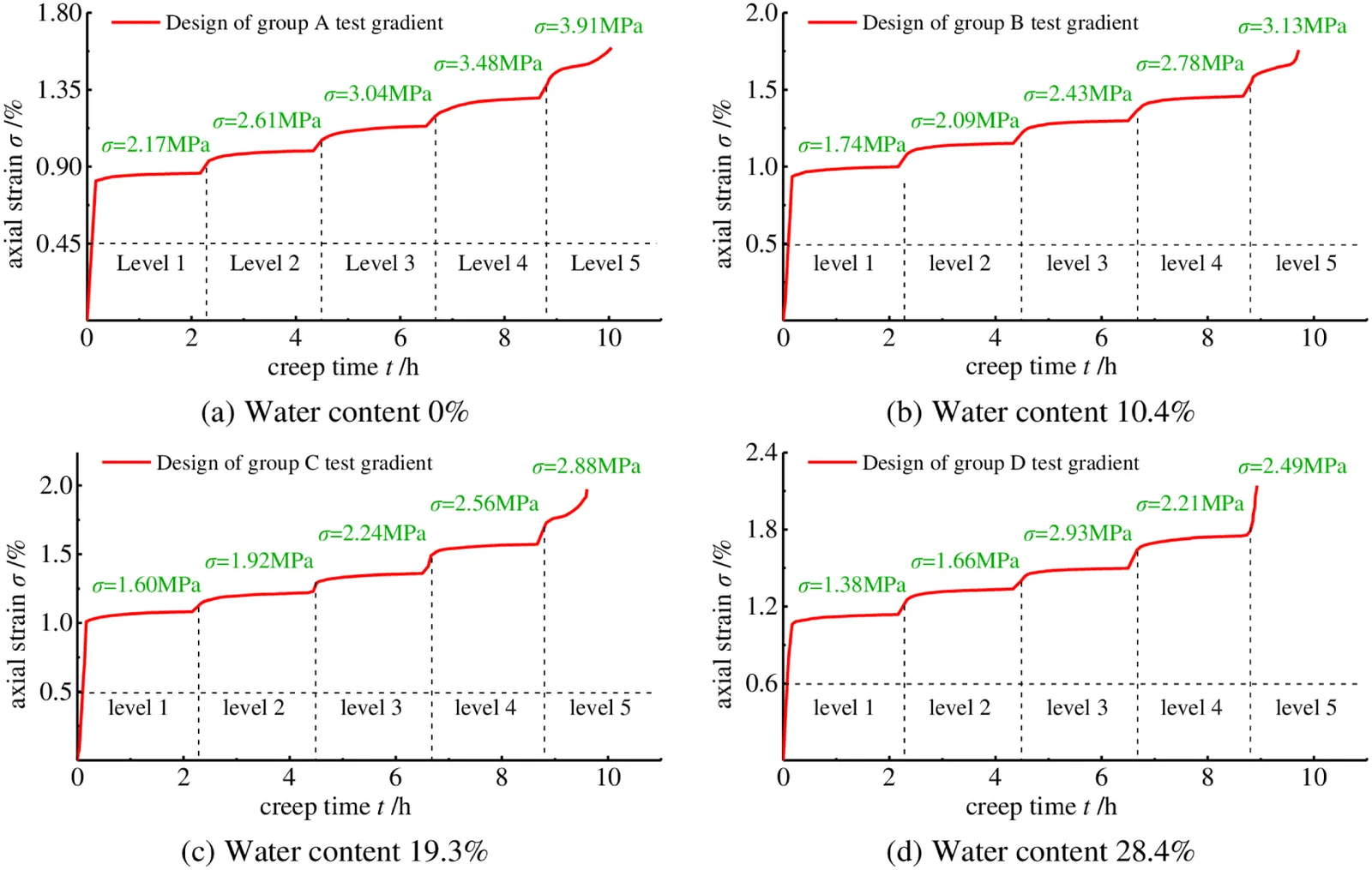
Axial strain curves of coal-rock specimens under multistage graded loading at different water contents. Reproduced from ref 56 (Guo, J.; Zhang, T.; Pan, H.; Wu, J. Experimental investigation of the creep damage evolution of coal rock around gas extraction boreholes at different water contents. *PLoS One* **2023**, *18* (2), e0278783), originally published under the Creative Commons Attribution License. License: https://creativecommons.org/licenses/by/4.0/.

In summary, borehole stability is positively correlated with coal mechanical strength and borehole support, while negatively correlated with in situ stress, mining-induced stress, gas pressure, drilling speed, drill pipe disturbance, borehole diameter, and other operational parameters. Notably, the instability of CMM drainage boreholes stems from the coupling of multiple factors. Under complex deep geological conditions, further research is required to identify primary and secondary influencing factors through multifield coupling experiments and numerical simulations, as well as their interaction mechanisms.

## 3 Instability Control Technologies for CMM Drainage Boreholes in Soft Coal Seams

The instability and failure of CMM drainage boreholes in soft coal seams are significant objective challenges caused by stratum mechanical properties. Based on the analysis of influencing factors for borehole instability, a prevention and control system for borehole instability is constructed in two stages: the borehole construction period and the service period.

### 3.1 Transient Instability Prevention Technologies during Borehole Construction Period

#### 3.1.1 Grouting Reinforcement

Owing to the abundant natural fractures and poor mechanical properties of deep coal, grouting is regarded as the most effective approach for reconstructing the physical properties of fragmented coal in underground strata. This method involves injecting liquid cement grout into the coal under controlled pump pressure to fill and solidify microfractures, fissures, and macroscopic joints, thereby achieving waterproofing and reinforcement effects.^100^ Zhai et al. proposed a regional solidification pore-forming technology. First, solidification holes around the target borehole are drilled, and then grout is injected under pressure into these holes. Once the grout fills and solidifies the surrounding fractures, the target borehole is then constructed. The grout can fill and consolidate fracture surfaces in the coal-rock mass, minimizing stress concentration under load.^41^ Jiang et al. proposed the “concentric ring” grouting reinforcement technology, involving first creating a “large-diameter” borehole, followed by reinforcement with high-strength cement-based grouting material. After the grout solidifies, a small-diameter borehole is drilled within the reinforced section, and finally, the borehole is sealed. The study found that the “concentric ring” grouting method can effectively solve the problems of easy collapse and stress concentration instability in the hole sealing section of soft coal seams, ensuring long-term and efficient sealing of gas drainage boreholes in soft coal seams.^32^ Li et al. studied simultaneous drilling-grouting technology, which utilizes foamed-concrete slurry for borehole wall protection during the drilling process. Results demonstrated that the selection of appropriate alternating spraying distance and nozzle diameter can enhance drilling efficiency, alleviate borehole collapse, and improve CMM drainage efficiency.^101^ Comparison of these three grouting reinforcement technologies is shown in Table 1.

**Table 1:** Comparison of Three Grouting Reinforcement Technologies

No.	Technologies	Advantages	Disadvantages
1	Regional solidification pore-forming technology^41^	Reinforces the borehole collar section; improves borehole success rate.	Long construction cycle; failure to resolve instability issues in the deep section of the borehole.
2	“Concentric ring” grouting reinforcement technology^32^	Reinforces the borehole collar section; improves gas drainage efficiency.	Long construction cycle; failure to resolve instability issues in the deep section of the borehole.
3	Drilling-grouting technology^101^	Inhibits borehole collapse in the deep section of the borehole; improves borehole success rate.	Simultaneous mode: nterference between grouting and cuttings discharge; the grout mixes with coal cuttings, solidifies, and blocks the cuttings discharge channel. Alternating mode: performance constrained by alternating nozzle spacing and nozzle inner diameter.^101^

A wide variety of grouting materials have been adopted for the grouting reinforcement technology for CMM drainage boreholes. Based on material composition, the current mainstream grouting materials can be classified into three categories: ordinary Portland cement-based grouting materials,^29^ modified cement-based grouting materials,^100,102^ and inorganic–organic composite grouting materials.^103^ Xu et al. proposed the development of nanofoam cement slurry materials featuring strong injectability, high mechanical strength, numerous open pores, and high permeability.^103^ These materials can not only stabilize boreholes but also promote CMM seepage and extend borehole service life, demonstrating broad application prospects in soft high-gas coal seam mining areas.

By effectively filling the fractures around the borehole in coal seams, grouting reinforcement technology reduces fracture instability and propagation, enhances coal interparticle cohesion and coal-rock mass stability, and ensures smooth drilling operations. However, this technology suffers from complex construction procedures, long implementation time, and high material costs. Additionally, the grouting process may impose adverse impacts on the underground environment. Most importantly, current grouting reinforcement research focuses more on borehole stability and sealing, with insufficient attention to the gas permeability of grouting materials.

#### 3.1.2 Specialized Drilling Techniques

Conventional drilling with hydraulic cuttings discharge, characterized by strong scouring action on the borehole wall, tends to induce severe borehole collapse and frequent pipe sticking incidents. Field practice has confirmed its inapplicability to soft coal seams.^104^ Currently, CMM drainage drilling operations in soft coal seams primarily employ three technologies: medium-pressure air drilling, high-speed spiral auger drilling, and air casing drilling.^30^ Air drilling technology causes minimal formation disturbance, but it suffers from severe dust pollution. Building upon this, scholars have developed air-assisted atomization drilling and air-foam drilling technologies, which can effectively mitigate dust pollution. Nevertheless, the formulations of atomizing liquids and foaming agents remain underdeveloped, and these technologies have not been widely implemented on a large scale.^91^ High-speed spiral drilling technology relies on the rotational action of spiral auger drill pipe flights for cuttings removal. With fast drilling speed and high cuttings removal efficiency, it effectively addresses cuttings accumulation and drill pipe sticking issues. However, plug-in drill pipes cause significant borehole deflection and severe disturbance to the borehole wall.^91,105^ During China’s 12th Five-Year Plan (2011–2015), aiming to integrate the advantages of air drilling and casing protection, researchers proposed air casing drilling technology, which has achieved favorable field application results. However, due to the single power head design, casing drilling tools lack free mobility during secondary drilling, presenting a risk of drill pipe burial.^106^ Additionally, since none of the above technologies enable precise borehole trajectory control, drill holes often deviate from the coal seam into the roof or floor, consequently failing to reach the designed depth.^30,106^ During China’s 13th Five-Year Plan (2016–2020), to address trajectory control issues, a pneumatic screw motor-powered directional drilling technology was successfully developed for the first time, enabling continuous directional drilling in soft coal seams.^107^ However, this technology faces challenges of low penetration rate and poor borehole wall stability in ultrasoft coal seams. Building on the limitations of air casing drilling technology and the application feasibility of pneumatic screw drilling tools, researchers developed double-pipe double-acting directional drilling technology.^30,106,108^ This technology, by integrating coaxial double-pipe double-acting operation, simultaneous casing running while drilling, pneumatic screw-driven precise orientation, and screen hole formation, enables precise control of large-diameter screen drilling trajectories and borehole quality. However, this technology involves complex operations for loading and unloading drilling tools during drilling, requires intermediate addition of casing and drill pipes, and entails long auxiliary operation time, all of which impact drilling efficiency.^108^ Integrating various drilling methods including air drilling, rotary drilling and directional drilling, Xi’an Research Institute has further proposed an internal controlled and guided rotary directional drilling technology. This technology employs a flexible internally controlled rotary directional drilling assembly, enabling real-time trajectory control while maintaining full-rotation cuttings removal.^109^ The principle feasibility of this technology has been demonstrated through prototype development and ground functional tests.^91^ The advantages and disadvantages of the aforementioned drilling technologies are summarized in Table 2.

**Table 2:** Comparison of Drilling Technologies in Soft Coal Seams

No.	Technologies	Advantages	Disadvantages
1	Medium-pressure air drilling technology^91,105^	Minimal disturbance to borehole wall.^91,105^	Severe dust pollution.^91^
2	Air atomization drilling technology^91^	Minimal disturbance to borehole wall; Effectively suppresses dust pollution.^91^	Atomization fluid formulation is not yet mature; Atomization performance depends on pressure stability.^91^
3	Air foam drilling technology^91^	Strong foam-coal seam compatibility; high cuttings-carrying capacity; Minimal disturbance to borehole wall.^91^	Foam formulation is not yet mature.^91^
4	High-speed spiral auger drilling technology^91,105^	High cuttings discharge efficiency; effectively prevents cuttings accumulation and pipe sticking.^105^	Manual connection of plug-in drill pipes required, resulting in high labor intensity; significant borehole oscillation, causing strong disturbance to the borehole wall.^91^
5	Pneumatic screw motor directional drilling technology^91,105^	Effectively controls borehole trajectory.^91,105^	The overall drilling efficiency is relatively low, and the hole-forming performance is unsatisfactory in extremely soft coal seams.^105^
6	Air-casing borehole protection drilling technology^91^	Effectively prevents borehole collapse; improves borehole completion rate.^91^	Complex operation; prolonged static setting of casing poses a risk of pipe burial; borehole trajectory cannot be precisely controlled.^91^
7	Double-pipe double-acting directional drilling technology^30,91,106,108^	Increases borehole depth; precisely controls borehole trajectory.^108^	Adding and removing double pipes during drilling increases labor intensity and operational complexity.^91,106,108^
8	Internal controlled and guided rotary directional drilling technology^91,109^	It integrates various drilling methods (including air drilling, rotary drilling, and directional drilling), leveraging their respective advantages.^91^	Still in the prototype testing and surface functional testing stage; lacks field industrial test verification.^91^

To address key technical challenges in soft coal seam drilling—such as borehole wall instability, difficulties in cuttings removal, and uncontrollable trajectories—scientific researchers and engineering technicians have conducted extensive studies on drilling technologies and drill tool designs. These efforts have achieved favorable application outcomes in enhancing drilling depth, improving drilling efficiency, increasing borehole quality rates, and controlling drilling trajectories in fractured soft coal seams. However, further improvements are still required. At present, drilling technologies for soft coal seams face challenges such as borehole wall instability, deformation, and inefficient cuttings removal. Owing to the intrinsic properties of soft coal seams, borehole collapse and gas burst frequently occur during drilling, significantly degrading drilling efficiency and borehole quality. Furthermore, existing drilling tools and processes exhibit significant limitations under complex geological conditions, particularly in terms of underground dust suppression, cooling and lubrication, and structural strength of drilling tools. Therefore, technical upgrades are required to improve drilling performance in soft coal seams.

### 3.2 Creep Damage Arresting Technologies during Borehole Service Period

#### 3.2.1 Screen Pipe Support

The stability degradation of coal-rock strata in soft coal seam renders the borehole prone to collapse, leading to blockage, failure, and severance of CMM drainage channels—thereby significantly compromising CMM drainage efficiency. Therefore, in soft outburst-prone coal seams, to ensure effective CMM drainage borehole, it is imperative to implement measures that prevent hole-wall collapse, which can obstruct CMM flow channels. Screen-pipe physical support technology is the preferred method for this purpose. Based on the differences in the pipe-running process, the technologies can be categorized into two types: open-hole screen pipe running technology and screen pipe running during drill pipe withdrawal.^110^

The open-hole screen pipe running technology involves withdrawing the drill pipe from the borehole after drilling is completed, followed by inserting a screen pipe into the open hole. This method offers advantages such as allowing a larger screen-pipe diameter and eliminating the need for specialized drilling tools. However, this method harbors several deficiencies. The perturbation induced by the drill pipe during withdrawal and the screen pipe during installation elevates the probability of borehole wall collapse. Moreover, the existence of coal debris and wall collapse within the borehole imposes limitations on the insertion depth of the screen pipe, frequently hindering its reaching the borehole bottom.^91,110–113^

To address the above-mentioned issues, in approximately 2012, screen pipe running during drill pipe withdrawal was developed. This technology operates by installing the screen pipe through the inner bore of the drill pipe without withdrawing the drill pipe. Axial thrust is applied to the screen pipe to deploy the open-close drill bit while withdrawing the drill pipe, and a suspension device mounted at the screen pipe’s leading end secures it within the borehole. After drill pipe retrieval, the screen pipe remains deployed within the borehole for structural protection and functions as a CMM drainage conduit, thereby enhancing CMM drainage efficiency. The primary supporting equipment for the screen pipe completion technology comprises large inner-bore drill pipes, open-close drill bits, borehole protection screen pipes, and suspension devices at the borehole bottom, enabling the achievement of enabling “drilling to the designed depth and installing the pipe to the bottom” with a screen pipe installation success rate exceeding 95%. This technology maintains compatibility with existing equipment, featuring simple and reliable operation. By installing the screen pipe through the drill pipe’s inner bore, it ensures insertion depth and installation success rate while enhancing CMM drainage efficiency and borehole service life.^91,111^ The comparison of screen pipe hole-protection technologies is shown in Table 3.

**Table 3:** Comparison of Screen Pipe Support Technologies

No.	Technologies	Advantages	Disadvantages
1	Open-hole screen pipe running technology^91,110–113^	Large screen pipe diameter.	Drill pipe withdrawal and screen pipe installation increase borehole wall collapse probability; Coal debris and collapsed material limit screen pipe insertion depth, frequently preventing it from reaching the borehole bottom.
2	Screen pipe running during drill pipe withdrawal technology^91,110,112^	Screen pipe can be run to borehole bottom, achieving full-hole section protection and extending the service life of boreholes.	Small screen pipe diameter; susceptible to crushing; screen holes prone to clogging.

Li et al. proposed an integrated drilling-protection-sealing technology, comprising directional drilling with a nitrogen slag discharge system, borehole protection, sealing, dust capturing, and safety assurance systems. Field applications in coal mines show that this technology effectively enhances CMM drainage in soft and broken coal seams. It has significantly improved the borehole construction success rate (from 45% to 96%) and protection success rate (from 43% to 100%). Additionally, the time required to eliminate coal seam outburst risk was reduced from 481 to 92 days, while CMM drainage efficiency increased from 27% to 82%.^83^

In addition, scholars have conducted extensive research on screen pipe materials, parameters, and internal support structures. They compared the compressive support performance of different screen pipe materials (e.g., PVC, PP, PE and ABS),^9,95^ designed internal support structure borehole-protection pipes^114^ and folded-expandable screen pipes,^91^ and investigated the effects of various screen pipe parameters (e.g., screen hole size, hole arrangement pattern, hole density) on borehole protection efficiency.^9,115–117^

In conclusion, screen pipe running during drill pipe withdrawal technology effectively addresses the issue of immediate borehole collapse in soft coal seams and significantly enhances CMM drainage efficiency. However, several limitations persist in practical applications. First, constrained by the inner diameter of the drill pipe, the screen pipe diameter is smaller than the borehole diameter, rendering the screen pipe unable to provide adequate borehole support and compromising CMM drainage efficiency. Second, larger screen hole diameters and greater screen hole density can enhance permeability and improve CMM drainage efficiency. However, they weaken the overall structural strength of the screen pipe, making it susceptible to plastic deformation or even flattening under stress. Additionally, when borehole wall collapse or damage occurs, the abundant coal debris and dust particles generated can easily clog the screen holes, increasing CMM flow resistance and negatively impacting CMM drainage.

#### 3.2.2 Backfilling with Porous Material

Cheng first proposed the method of “porous material backfilling hole protection”.^118^ During the drill pipe withdrawal stage, foam concrete, prepared via a physical foaming method, was injected into the borehole through a hollow drill pipe to backfill the drainage section. Once the porous material reaches the designated position within the borehole, it solidifies and provides structural support to prevent collapse. Ling conducted studies on the application of foam concrete prepared via a chemical foaming method using foaming agents for borehole support.^119^ The primary support targets were the fractured and plastic zones within boreholes prone to collapse. It was found that prior to borehole collapse, the barrier effect of foam concrete on CMM seepage resulted in a 20.48% decrease in average mixed CMM flow rate and a 21.54% decrease in average pure CMM flow rate in test boreholes relative to control ones. Following borehole collapse, the supporting effect of foam concrete led to an 81.74% increase in average mixed CMM flow rate and a 78.27% increase in average pure CMM flow rate in test boreholes. The above two studies were based on porous materials for drilling support, and the feasibility of the technology is proved by field tests. In recent years, Wang and Zhang et al. have proposed a method of collaborative support technology with porous materials and sieve tubes.^91,96^ Among them, Zhang et al. further developed an environmentally friendly high-strength, high-permeability foam concrete.^96^ Field tests demonstrated that, compared with boreholes using only screen pipes, test boreholes showed 81.74% higher mixed CMM flow and 78.27% higher pure CMM flow after borehole collapse, effectively ensuring unobstructed drainage channels. The comparison of borehole filling and protection technologies is shown in Table 4.

**Table 4:** Comparison of Backfilling Hole-Protection Technologies

No.	Technologies	Advantages	Disadvantages
1	Grouting hole-protection technology^118,119^	The borehole wall can be directly supported while gas migration channels are reserved.	The grouting material has poor permeability and hinders the transmission of drainage negative pressure to a certain extent.
2	Cooperative protection of screen pipe and grouting technology^91,96^	Better anticollapse capacity than single grouting protection technology.	The grouting material has poor permeability and hinders the transmission of drainage negative pressure to a certain extent; complex construction procedures and higher cost.

The porous material filling hole protection technology demonstrates significant advantages in preventing borehole collapse. However, this technology exhibits certain limitations during the early stages of CMM drainage. During the period when the borehole has not collapsed, the porosity of the porous material is inferior to that of the screen pipe, which may impede negative pressure transmission and CMM drainage. Consequently, this results in a less effective CMM drainage compared to the screen pipe hole protection technology. Currently, a more feasible solution involves using screen pipes in conjunction with porous material to address this issue. To further enhance the application of this technology in CMM drainage from soft coal seams, future research directions should focus on the following two key aspects: the CMM permeability of porous materials and their compressive performance. Meanwhile, during the material research and development process, the environmental impact and cost–effectiveness of the materials should also be comprehensively considered to ensure the sustainable development and wide application of this technology.

#### 3.2.3 Intelligent Monitoring

Borehole monitoring is a prerequisite for addressing borehole instability. It enables the acquisition of real–time status information on boreholes, thus effectively preventing borehole collapse. Currently, borehole deformation can be assessed using various methods, including drilling flushing fluid method,^120^ drilling cuttings method,^121^ logging technology,^122^ coal core method,^62^ acoustic wave method,^123^ endoscopic method,^124^ and changes in CMM drainage parameters (e.g., CMM flow and negative pressure).^78,125^ However, with the increasing demand for borehole refinement, traditional borehole collapse monitoring technologies are unable to meet the goals of refined borehole monitoring.

In recent years, with the continuous advancement of intelligent technologies, real–time monitoring of CMM drainage boreholes has emerged as a research hotspot. Zhao et al. used resistance strain gauges to monitor borehole strain and identified the transition of the strain curve from rapid growth to decay as a precursor to borehole collapse.^59^ Niu et al. employed a soft elastic capsule to determine the degree of destruction of the borehole by monitoring the relative pressure changes within the capsule.^60,61^ Cheng et al. arranged fiber-optic grating sensors along the borehole and located collapse positions and estimated collapsed coal mass by measuring the center wavelength shift of the gratings, achieving point monitoring of borehole collapse.^120^ Xiao et al. employed Brillouin Optical Time Domain Analysis Technique (BOTDA) and back-calculated the borehole plugging rate by monitoring the continuous strain distribution along the fiber, enabling full-hole continuous quantitative evaluation of collapse severity.^62^ The four aforementioned monitoring techniques show an evolutionary trend in spatial coverage: point sensing, cross-section sensing, quasi-distributed sensing, and fully continuous distributed sensing. This development is accompanied by gradual improvements in monitoring accuracy and spatial localization performance. A comparison of intelligent monitoring technologies for borehole instability is presented in Table 5.

**Table 5:** Comparison of Intelligent Monitoring Technologies for Borehole Instability.

No.	Technologies	Advantages	Disadvantages
1	Resistance strain gauge monitoring technology^59,92^	High measurement accuracy; dynamic evolution characteristics of borehole instability can be captured.	Active sensing; long transmission cables are susceptible to underground temperature fluctuation and electromagnetic interference, resulting in poor stability for long-term monitoring.
2	Soft elastic capsulemonitoring technology^60,61^	Simple structure, low cost and convenient for field popularization.	Segmented monitoring mode with low spatial resolution.
3	Fiber-optic grating (FBG) monitoring technology^120^	Point-type monitoring; passive fiber sensor, antielectromagnetic interference, corrosion and moisture resistant.	Blind areas exist between measuring points; matched demodulation equipment is required, leading to high system costs.
4	BOTDA technology^62^	Full-length continuous monitoring; quantitative calculation of collapse degree; antielectromagnetic interference, corrosion and moisture resistant.	Matched optical fiber demodulation equipment is required, leading to high system cost.

Due to the inability to accurately predict the locations of borehole collapse, the full-hole section protection method is used in soft coal seams, which results in a substantial waste of human and material resources. In response to this technical challenge, scholars have proposed borehole monitoring systems, which have laid a theoretical foundation for the precise support of CMM drainage boreholes. However, the relevant technologies are currently primarily in the laboratory research and development stage. The geological conditions and mining environments at actual coal mine sites are highly complex, with significant differences from laboratory simulations. Moreover, the system installation is complicated and costly, requiring a high level of professional expertise from operators, which may limit their further promotion and application.

## 4 Technical Bottlenecks Analysis and Future Prospects

This review systematically reveals the instability mechanisms of CMM drainage boreholes and expounds the influencing factors of borehole instability from the aspects of geological structures, surrounding rock stress, coal mechanical properties, engineering factors, and environmental factors. A comprehensive review of existing borehole stability control technologies reveals that the application of chemical grouting, drilling techniques, screen pipe physical support, porous media backfilling, and monitoring technologies could significantly extend the service life of boreholes and enhance CMM drainage efficiency. However, several technical bottlenecks still exist in the current research. Given these challenges, and focusing on the technical limitations while combining them with the needs of engineering practice, this review identifies targeted future research directions, with the aim of serving as a reference for subsequent studies.

### 4.1 Technical Bottlenecks

#### 4.1.1 Ambiguity in Deep Multi-Field Coupling Instability Mechanisms

The instability of CMM drainage boreholes is a complex mechanical problem that involves the coupling of multiple physical fields, including mechanics, thermodynamics, and seepage. The heterogeneity and anisotropy of coal-rock, coupled with the effects of high in situ stress, high gas pressure, and asymmetric confining pressure in deep formations, further complicate this issue. However, existing theoretical analyses, numerical simulations, and experimental methods still have limitations in addressing such complex problems and cannot fully and accurately reproduce the entire process of borehole instability. Therefore, scholars are compelled to conduct relevant research using simplified models. While this simplification aids in analyzing the problem to some extent, it also means that the dynamic instability mechanisms of boreholes under complex deep geological conditions remain incompletely understood.

#### 4.1.2 Maturity Shortage of Real–Time Monitoring and Early Warning Technologies

During CMM drainage in soft coal seams, borehole instability is a common issue. Accurately locating the instability position is a critical step for dredging the borehole and restoring its drainage function. In recent years, novel sensor technologies such as fiber Bragg gratings have been gradually applied to borehole stability monitoring. Nevertheless, this technology is still at the experimental-research stage and has not been fully mature. Its stability and reliability need further verification. Meanwhile, under the complex geological conditions of coal mines, the installation and maintenance of fiber Bragg grating sensors encounter considerable challenges. Such work demands professional technicians and involves high costs. Collectively, these factors may restrict its large-scale popularization and field application.

#### 4.1.3 Ambiguity in Permeability–Strength Balance Mechanism of Screen Pipes

Current research on the balance mechanism between the permeability and strength of screen pipes is insufficient. The structural design and material properties of screen pipes are key factors that determine their permeability, strength, and antideformation capabilities. At present, the design of screen pipes is primarily based on idealized mechanical models, which do not fully account for the complexity and dynamic variability of actual working conditions in different coal mines. In the pursuit of CMM drainage efficiency, the permeability of the screen pipe is often overemphasized, neglecting the optimal balance between permeability, strength, and antideformation capabilities.

#### 4.1.4 Inadequate Permeability of Filling Borehole-Protection Materials

Some scholars have conducted relevant research on high-permeability filling borehole-protection materials, with foam concrete being the primary research object. Existing results indicate that although the permeability of foam concrete is higher than that of deep soft coal seams, it still imposes a certain hindrance to the transmission of extraction negative pressure. In some experiments, it was found that the extraction effect under this borehole-protection method was unsatisfactory compared to that before borehole collapse. This suggests that filling borehole-protection materials with both adequate strength and high permeability still require further development.

#### 4.1.5 Maturity Shortage of Borehole Dredging Technologies

Compared to redrilling, unblocking clogged boreholes to restore CMM drainage functions should be more cost-effective and efficient. However, in coal mine sites, secondary drilling is often the preferred choice. The main reasons are as follows: existing unblocking processes rely heavily on manual experience, necessitating repeated attempts to locate the blockage, which is time-consuming and has a low success rate. Traditional unblocking equipment is bulky, making it difficult to operate in the narrow underground spaces and potentially causing secondary hole collapse. Due to the uncertain efficiency of unblocking, the total time and labor costs may exceed those of direct drilling, thereby forcing the site to abandon dredging efforts.

### 4.2 Future Prospects

Based on technical bottlenecks and engineering requirements, future research should focus on the following directions:

#### 4.2.1 Multi Field Coupling Mechanism for Deep Borehole Instability

To accurately characterize complex deep geological conditions, a coupled numerical model considering stress, gas seepage, thermal effect, hydraulic effect and damage evolution shall be established. High in situ stress, high geothermal temperature, high gas pressure, low permeability as well as asymmetric confining pressure shall also be taken into account in this model to reveal the coupled evolution characteristics of multiple physical fields governing deep borehole instability.

#### 4.2.2 Intelligent Monitoring and Early Warning Systems for Borehole Stability

Intelligent monitoring and early warning systems for borehole stability shall be developed and integrated with the existing CMM drainage parameter monitoring equipment in coal mines. Advanced data processing and analysis technologies shall be utilized to perform comprehensive analysis and data mining on multiparameter data sets. The device can realize early warning of borehole instability and trigger timely prereinforcement measures. Meanwhile, for boreholes that have already suffered instability, it can accurately identify the location and degree of instability, providing a scientific basis for borehole dredging and repair.

#### 4.2.3 High-Strength and Anticlogging Borehole-Protection Screen Pipes

Corrosion-resistant, high-strength and low-cost alloys or composite materials shall be developed to improve the compressive capacity and durability of screen pipes. In addition, multilayer or cross-support configurations shall be adopted to optimize internal support designs and enhance the internal stability of screen pipes under high-pressure conditions. Simultaneously, the shape, size and arrangement of screen holes shall be optimized to expand the CMM gas-permeable area and reduce the clogging probability.

#### 4.2.4 Porous Self-Expanding Borehole-Protection Materials

Porous self-expanding borehole-protection materials with diverse compressive strength and permeability shall be developed to satisfy the demands of different geological conditions. Injectable during drill-bit withdrawal, such materials should rapidly achieve their designed compressive strength and possess porous permeable properties, providing migration channels for CMM. Owing to the self-expansion performance, the materials should support the borehole and mitigate borehole instability.

#### 4.2.5 Inversion of Performance Indicators of Borehole-Protection Materials under Different Working Conditions

Performance requirements for borehole-protection materials differ under various coal-seam working conditions. To realize efficient CMM drainage and avoid resource waste, it is urgent to carry out inversion studies on key performance indicators (e.g., strength, permeability, fluidity) of borehole-protection materials based on actual coal-seam conditions. Such research can, on the one hand, provide theoretical guidance for the development of borehole-protection materials, and on the other hand, help coal mines select suitable materials for specific working conditions.

#### 4.2.6 Miniature Intelligent Borehole Dredging Technologies and Equipment

Miniature intelligent borehole dredging technologies and equipment suitable for underground operations shall be developed. The equipment shall feature flexible operation and occupy little underground space. Supported by the real-time borehole monitoring system, it shall timely remove in-hole collapse debris at the onset of borehole collapse, so as to prevent continuous accumulation of blockages and avoid further deterioration of borehole clogging.

## References

[1] National Bureau of Statistics of China . Statistical Communique on the 2025 National Economic and Social Development of the People’s Republic of China, 2026. https://www.stats.gov.cn/sj/zxfb/202602/t20260228_1962662.html (accessed Aug 10, 2026).

[2] Cheng X., Cheng C., Xiao L., Ma X. (2024). Response surface analysis
on multiple parameter effects on borehole gas extraction efficiency. Processes.

[3] Yi M., Wang L., Hao C., Liu Q., Wang Z. (2021). Method for
designing the optimal sealing depth in methane drainage boreholes
to realize efficient drainage. Int. J. Coal
Sci. Tech..

[4] Zheng C., Jiang B., Xue S., Chen Z., Li H. (2019). Coalbed methane
emissions and drainage methods in underground mining for mining safety
and environmental benefits: a review. Process
Saf. Environ. Prot..

[5] Zhang L., Zhang H., Guo H. (2017). A case study of gas
drainage to low
permeability coal seam. Int. J. Min. Sci. Technol..

[6] Wang W., Yang W., Zhang Z., Shi W. (2026). Multifactor analysis
and dominant factor identification for coal seam gas drainage borehole
instability. Sci. Rep..

[7] Ren Z., Wang H. (2023). Gas migration around
large diameter borehole with the protective
screen pipe in fractured soft coal seam. ACS
Omega.

[8] Wang F., Ren T., Tu S., Hungerford F., Aziz N. (2012). Implementation of underground
longhole directional drilling technology for greenhouse gas mitigation
in chinese coal mines. Int. J. Greenhouse Gas
Control.

[9] He S., Ou S., Huang F., Jin L., Ma Y., Chen T. (2022). Borehole protection
technology of screen pipes for gas drainage boreholes in soft coal
seams. Energies.

[10] Chen B., Barboza B. R., Sun Y., Bai J., Thomas H. R., Dutko M., Cottrell M., Li C. (2022). A review of
hydraulic
fracturing simulation. Arch Computat Methods
Eng..

[11] Liu Y., Xia B., Liu X. (2015). A novel method of orienting hydraulic
fractures in
coal mines and its mechanism of intensified conduction. J. Nat. Gas Sci. Eng..

[12] Niu X., Pang D., Liu H., Zhang Y., Cheng G., Cao J., Zhao Y. (2023). Gas extraction mechanism and effect of ultra-high-pressure
hydraulic slotting technology: a case study in renlou coal mine. Nat. Resour. Res..

[13] Wei G., Wen H., Deng J., Li Z., Fan S., Lei C., Liu M., Ren L. (2021). Enhanced coalbed
permeability and methane recovery
via hydraulic slotting combined with liquid CO2 injection. Process Saf. Environ. Prot..

[14] Liu T., Lin B., Fu X., Zhao Y., Gao Y., Yang W. (2021). Modeling coupled
gas flow and geomechanics process in stimulated coal seam by hydraulic
flushing. Int. J. Rock Mech. Min. Sci..

[15] Yang L., Fan C., Wen H., Luo M., Sun H., Jia C. (2023). An improved
gas-liquid-solid coupling model with plastic failure for hydraulic
flushing in gassy coal seam and application in borehole arrangement. Phys. Fluids.

[16] Zhang H., Cheng Y., Liu Q., Yuan L., Dong J., Wang L., Qi Y., Wang W. (2017). A novel in-seam borehole
hydraulic flushing gas extraction technology in the heading face:
enhanced permeability mechanism, gas flow characteristics, and application. J. Nat. Gas Sci. Eng..

[17] Zhang R., Wang P., Cheng Y., Shu L., Liu Y., Zhang Z., Zhou H., Wang L. (2022). A new technology to
enhance gas drainage in the composite coal seam with tectonic coal
sublayer. J. Nat. Gas Sci. Eng..

[18] Cheng X., Wen H., Fan S., Chen J., Zhai X., Yu Z., Tong X., Lei C., Xu Y., Cheng B. (2021). Liquid CO2 high-pressure
fracturing of coal seams and gas extraction
engineering tests using crossing holes: a case study of panji coal
mine no. 3, huainan, china. Int. J. Energy Res..

[19] Ren F., Zhang D., Wang C., Lu X., Wang P., Zhang Y., Xiong Z. (2025). Influence of liquid
CO2 phase transition
blasting on hydraulic fracturing in combined fracturing conditions. Phys. Fluids.

[20] Guo D., Lv P., Zhao J., Zhang C. (2020). Research progress on permeability
improvement mechanisms and technologies of coalbed deep-hole cumulative
blasting. Int. J. Coal Sci. Tech..

[21] Zhao Z., Liu P., Li Q., Nie B., Zhao Y., Song J., Bao G., He H., Liu W., Sun L. (2025). Enhancing coalbed methane
recovery using high power ultrasonic excitation: a nano-micro-to-engineering
scale study. Energy.

[22] Chen X., Shi B., Cao Y., Long P., Qi Y., Li L. (2024). Study on the
wettability and methane desorption characteristics of coal under high
pressure with different surfactants. ACS Omega.

[23] Liu S., Sun H., Zhang D., Yang K., Li X., Wang D., Li Y. (2023). Experimental study of effect of liquid nitrogen cold soaking on coal
pore structure and fractal characteristics. Energy.

[24] Wang Y., Yan W., Ren Z., Yan Z., Liu Z., Zhang H. (2020). Investigation
of large-diameter borehole for enhancing permeability and gas extraction
in soft coal seam. Geofluids.

[25] Wang Y., Zhao X., Wang P., Chen Y. (2023). Gas evolution principle
during gas drainage from a drill hole along the coal seam and reasonable
borehole layout spacing. ACS Omega.

[26] Wei P., Huang C., Li X., Peng S., Lu Y. (2019). Numerical
simulation of boreholes for gas extraction and effective range of
gas extraction in soft coal seams. Energy Sci.
Eng..

[27] Zhang J., Han Z., Chen T., Yao N., Yang X., Chen C., Cai J. (2024). A numerical simulation of the coal dust migration law in directional
air drilling in a broken soft coal seam. Processes.

[28] Zhou A., Li J., Gong W., Wang K., Du C. (2023). Theoretical and numerical
study on the contribution of multi-hole arrangement to coalbed methane
extraction. Energy.

[29] Bao R., Zhou F., Shang H., Song S. (2024). Study on key grouting
blocking parameters of gas drainage boreholes in soft coal seams. Heliyon.

[30] Nie C., Wang Y., Yao Y., Hong J. (2022). Powder discharge characteristics
of pneumatic double pipe directional drilling in broken soft coal
seams and its application. Coal Geol. Explor..

[31] Gao Y., Ren J. (2022). Study on the effect
of borehole size on gas extraction borehole strength
and failure mode. ACS Omega.

[32] Jiang L., Bao R., Lei C. (2024). Expansion
characteristics and creep test of new curing
expansion material for gas extraction boreholes. Processes.

[33] Liu C., Xiao X., Yang L., Yang K. (2013). Failure analysis of
the methane drainage borehole in the weak coal seam. Dis. Adv..

[34] Hao F., Sun L., Zhao F. (2020). Borehole diameter shrinkage rule considering rheological
properties and its effect on gas extraction. PLoS One.

[35] Xie H., Gao M., Zhang R., Peng G., Wang W., Li A. (2019). Study on the
mechanical properties and mechanical response of coal mining at 1000
m or deeper. Rock Mech. Rock Eng..

[36] Du X., Xue J., Yu L., Lei W., Ma H., Cao C. -R., Shu C. -M., Li Y. (2024). Coal damage and energy characteristics
during shallow mining to deep mining. Energy.

[37] Li X., Wang E., Li Z., Liu Z., Song D., Qiu L. (2016). Rock burst monitoring by integrated
microseismic and electromagnetic
radiation methods. Rock Mech. Rock Eng..

[38] Li X., Chen S., Wang E., Li Z. (2021). Rockburst mechanism
in coal rock with structural surface and the microseismic (ms). and
electromagnetic radiation (emr). response. Eng.
Failure Anal..

[39] Chen Y., Chen C., Liu Z., Hu Z., Wang X. (2015). The methodology
function of citespace mapping knowledge domains. Studies in Science of Science..

[40] Liu C., Zhou F., Wang S., Liu Y., Xiao X. (2012). Study on horizontal
deformation of vertical borehole during two coal seams exploitation. Dis. Adv..

[41] Zhai C., Li Q., Sun C., Ni G., Yang W. (2012). Analysis on borehole
instability and control method of pore-forming of hydraulic fracturing
in soft coal seam. J. China Coal Soc..

[42] Wang Z., Liang Y., Jin H. (2008). Analysis of
mechanics conditions
for instability of outburst-preventing borehole. J. Mining Saf. Eng..

[43] Yao X., Cheng G., Shi B. (2010). Analysis on gas extraction drilling
instability and control method of pore-forming in deep surrounding-rock
with weak structure. J. China Coal Soc..

[44] Duan K., Wu W., Kwok C. Y. (2018). Discrete
element modelling of stress-induced instability
of directional drilling boreholes in anisotropic rock. Tunnell. Underground Space Technol..

[45] Hashemi S. S., Melkoumian N. (2016). Effect of
different stress path regimes on borehole
instability in poorly cemented granular formations. J. Pet. Sci. Eng..

[46] Liu Q., Cheng Y., Jin K., Tu Q., Zhao W., Zhang R. (2017). Effect of confining pressure unloading on strength reduction of soft
coal in borehole stability analysis. Environ.
Earth Sci..

[47] Zhai C., Xu Y., Xiang X., Yu X., Zou Q., Zhong C. (2015). A novel active
prevention technology for borehole instability under the influence
of mining activities. J. Nat. Gas Sci. Eng..

[48] Cheng H., Zhang N., Yang Y., Dong Y., Peng W. (2018). 3-d dynamic
evolution analysis of coal-rock damaged field and gas seepage field
during the gas extraction process. J. Nat. Gas
Sci. Eng..

[49] Wang Z., Sun Y. (2018). Swelling pressure of double-expansive material and its active support
effect for coal seam gas drainage borehole. Adv. Mater. Sci. Eng..

[50] Wang Z., Sun Y., Li Z., Wang Y., You Z. (2022). Multiphysics responses
of coal seam gas extraction with borehole sealed by active support
sealing method and its applications. J. Nat.
Gas Sci. Eng..

[51] Xue Y., Ranjith P. G., Dang F., Liu J., Wang S., Xia T., Gao Y. (2020). Analysis of deformation,
permeability and energy evolution
characteristics of coal mass around borehole after excavation. Nat. Resour. Res..

[52] Qiu Y., Ma T., Liu J., Fadhel A. M., Peng N., Xu H., Ranjith P. G. (2025). Thermal-hydro-mechanical
coupled dual-medium model
of inclined wellbore in fractured anisotropic formations. Geoenergy Sci. Eng..

[53] Lu J., Yin G., Gao H., Li X., Zhang D., Deng B., Wu M., Li M. (2020). True triaxial experimental study of disturbed compound
dynamic disaster in deep underground coal mine. Rock Mech. Rock Eng..

[54] Xu C., Qin L., Wang K., Wu S., Zhang R., Guo H., Shu L. (2023). Deformation and failure
characteristics of gas drainage drilling-reaming
coal mass in non-uniform stress field. J. China
Coal Soc..

[55] Wang X., Ma H., Qi X., Gao K., Li S., Yang X. (2022). Experimental
study on the effect of high-temperature oxidation coal mechanical
characteristics. PLoS One.

[56] Guo J., Zhang T., Pan H., Wu J. (2023). Experimental investigation
of the creep damage evolution of coal rock around gas extraction boreholes
at different water contents. PLoS One.

[57] Zhang H., Zhang T. (2024). Study on progressive damage and deformation
law of coal body around
borehole under different moisture states. Environ.
Earth Sci..

[58] Zhang T., Ling Z., Pang M., Meng Y. (2021). Experimental study
of creep acoustic emission characteristics of coal bodies around boreholes
under different moisture contents. Energies.

[59] Zhao H., Li J., Liu Y., Wang Y., Wang T., Cheng H. (2020). Experimental
and measured research on three-dimensional deformation law of gas
drainage borehole in coal seam. Int. J. Min.
Sci. Technol..

[60] Niu Y., Song X., Li Z., Wang E., Liu Q., Zhang X., Cai G., Zhang Q. (2020). Experimental study
and field verification of stability monitoring of gas drainage borehole
in mining coal seam. J. Pet. Sci. Eng..

[61] Niu Y., Zhang X., Wang E., Li Z., Cheng Z., Duan X., Li H., Wei Y., Qian J., Cai G. (2020). A new method of monitoring
the stability of boreholes
for methane drainage from coal seams. Measurement.

[62] Xiao P., Huang X., Zhang C., Liu X., Li B., Chen L., Chen Z., Cheng R., Zhao Y. (2024). Research on
the construction and verification of evaluation model for borehole
collapse in gas extraction. Fuel.

[63] Pan H., Shuai J., Li J., Li H., Zhang H., Zhang T., Ji X. (2025). Analysis of deformation
behavior
characteristics of double-prevention boreholes based on pearson correlation
coefficient. Rock Mech. Rock Eng..

[64] Cheng C., Hu Q., Luo Y., Wang B., Tao R., Sun Y. (2025). Experimental
study of deformation evolution around the gas extraction borehole
in a composite stratum. Fuel.

[65] Jia T., Feng Z., Wei G., Ju Y. (2018). Shear deformation of
fold structures in coal measure strata and coal-gas outbursts: constraint
and mechanism. Energy Explor. Exploit..

[66] Liu Y., Ba Q., Shen K., Jin N. (2020). Research and application of water
jet and cooperative repair technology in gas drainage. Min. Saf. Environ. Proteet.

[67] Shen R., Qu P., Yang H. (2010). Advancement
and development of coal bed wellbore stability
technology. Drill. Pet. Tech..

[68] Zhang B., Bai H., Zhang K. (2016). Seepage characteristics
of collapse column fillings. Int. J. Min. Sci.
Technol..

[69] Yang T., Xu G., Chen K., Sun G., Dang B., Liu M. (2023). Characteristics
and evolution of karst collapse columns in the huainan coalfield. Sci. Total Environ..

[70] Cao Z., Zhang S., Li Z., Du F, Huang C., Wang W. (2024). Numerical research
on disastrous mechanism of seepage instability
of karst collapse column considering variable mass effect. Sci. Rep.

[71] Zhao J., Wang X., Bai J., Wang G., Chen D., Li G. (2024). Study on instability mechanisms and
control of coal pillar spalling
and coal crumbs leakage during working face crossing faults. Sci. Rep..

[72] Kong X., Zhao T., Cai Y., He D. (2024). Numerical
multifield
coupling model of stress evolution and gas migration: application
of disaster prediction and mining sustainability development. Sustainability.

[73] Xie H., Li C., He Z., Li C., Lu Y., Zhang R., Gao M., Gao F. (2021). Experimental study on rock mechanical behavior retaining
the in situ geological conditions at different depths. Int. J. Rock Mech. Min. Sci..

[74] Zhang X., Wang W., Cai H. (2021). Study on influencing
factors of deformation
and instability of deep coal seam drainage borehole. Coal Sci. Technol..

[75] Sun X., Ran Q., Liu H., Ning Y., Ma T. (2023). Characteristics of
stress-displacement-fracture multi-field evolution around gas extraction
borehole. Energies.

[76] Gao B., Wang Z., Li H., Su C. (2018). Experimental study
on the effect of outburst-proneness of coal by gas pressure. J. China Coal Soc..

[77] Gao B., Qian Y., Chen L., Wang Z., Lyu P. (2018). Study on gas
pressure affected to bump potential of coal sample. Coal Sci. Technol..

[78] Zhang X., Shen S., Feng X., Ming Y., Liu J. (2021). Influence
of deformation and instability of borehole on gas extraction in deep
mining soft coal seam. Adv. Civil Eng..

[79] Wang Y., Li C., Hao M., Zhang H., Sun X. (2022). Damage characteristics
of pulverized coal under different gas pressures in coal and gas outbursts. Energy Sources, Part A.

[80] Wang L., Chen L., Liu H., Zhu C., Li S., Fan H., Zhang S., Wang A. (2023). Dynamic behaviors
and deterioration
characteristics of coal under different initial gas pressures. Rock Soil Mech..

[81] Liu C., Zhou F., Kang J., Xia T. (2016). Application of a non-linear
viscoelastic-plastic rheological model of soft coal on borehole stability. J. Nat. Gas Sci. Eng..

[82] Zhang G., Zhang S., Jia W., Zhang X., Zhang Y., Lian H., Lian X., Fang H. (2023). Grouting solidification
technology for fractured soft coal seams and its application in coalbed
methane (coal mine gas). extraction. Front.
Energy Res..

[83] Li H., Wang W., Liu Y., Ma J., Gao H. (2020). An integrated
drilling, protection and sealing technology for improving the gas
drainage effect in soft coal seams. Energy Rep..

[84] Xu Y., Du Y., Zhang K., Pang X., Xu Y. (2023). Research and application
of different coal wall spalling forms. Earth
Sci..

[85] Wu D., Li B., Wu J., Hu G., Gao X., Lu J. (2023). Influence
of mineral composition on rock mechanics properties and brittleness
evaluation of surrounding rocks in soft coal seams. ACS Omega.

[86] Zheng J., Wang L. (2024). Experimental study
on creep loading of porous coal under different
influencing factors. Facta Univ., Ser. Mechan.
Eng..

[87] Wang Y., Xu M., Kong D., Wu G., Shang Y., Chen L., Liu Y. (2023). Study on destabilization failure characteristics and energy evolution
law of drilling holes. Energy Sci. Eng..

[88] Wang Y., Yu Z., Guo J., Du K., Ma D., Zhao A. (2024). Mechanism
and application of drill pipe bending induced borehole collapse in
soft coal seam drilling. Energy Sci. Eng..

[89] Wang L., Wang D., Chen X., Min R. (2024). Promoting gas extraction
technology with screen pipe for long borehole protection in soft seam. Processes.

[90] Yang S., Wen G., Yan F., Zhang M., Zhao X. (2020). Evaluating the maximum
rate of penetration for drilling borehole in soft coal seam. Energy Sci. Eng..

[91] Wang L., Yao N., Yao Y., Wang Y., Zhang J., Fang J., Wei H. (2021). Research progress
of drilling and borehole completion technologies
in broken soft coal seam in underground coal mine. Coal Geol. Explor..

[92] Zhao H., Zhang M., Zhang H., Wang H., Li F. (2018). Development
and application of stability dynamic monitoring and measuring device
to gas drainage borehole. Coal Sci. Technol..

[93] Cao G., Zhang C., Li Z., Ma H., Cai D., Zhou X., Zhang X., Bai L., Zhang P., Zhao J. (2025). Design and evaluation of drilling
fluid systems for wellbore stabilization
during drilling in deep coalbed gas reservoirs in the ordos basin. Processes.

[94] Liu X., Dang B., Li L., Bai S., Tan Q., Sun Q. (2026). Research on wellbore stability prediction of deep coalbed methane
under multifactor influences. Appl. Sci..

[95] Liu C., Man Z., Tian J. (2025). Failure mechanisms
of boreholes in soft coal seams:
comparative analysis of protective sieve pipes. Int. J. Geomech..

[96] Zhang X., Wang Y., Gao J., Han L., Ge X., Yang M., Wang S. (2025). Development and application of high-performance
drilling hole protection materials. Sci. Rep..

[97] Yuan R., Chen C., Wei X., Li X. (2019). Heat–fluid–solid
coupling model for gas-bearing coal seam and numerical modeling on
gas drainage promotion by heat injection. Int.
J. Coal Sci. Tech..

[98] Cao N., Jing P., Huo Z., Liang Y., Zhang L. (2024). Simulation
study of coal seam gas extraction characteristics based on coal permeability
evolution model under thermal effect. ACS Omega.

[99] Gao M., Liu Y. (2021). Experimental
study on the influence of moisture content on the mechanical
properties of coal. Geofluids.

[100] Li B., Zhang J., Liu Y., Qu L., Liu Q., Sun Y., Xu G. (2022). Interfacial porosity
model and modification mechanism
of broken coal grouting: a theoretical and experimental study. Surf. Interfaces.

[101] Li D. (2016). A new technology for
the drilling of long boreholes for gas drainage
in a soft coal seam. J. Pet. Sci. Eng..

[102] Sha F., Fan R., Gu S., Xi M. (2023). Strengthening effect
of sulphoaluminate cementitious grouting material for water-bearing
broken rocky stratum. Constr. Build. Mater..

[103] Xu C., Li H., Lu Y., Lu J., Shi S. (2022). Research status
of borehole instability characteristics and control technology for
gas extraction in soft coal seam. Min. Saf.
Environ. Prot..

[104] Yin X., Liu J., Ji Q. (2012). Medium wind pressure air drilling
technique and equipments in soft coal seam. Saf. Coal Mines.

[105] Yao N., Wang Y., Yao Y., Song H., Wang L., Peng T., Sun X. (2020). Progress of drilling technologies
and equipments for complicated geologicalconditions in underground
coal mines in china. Coal Geol. Explor..

[106] Wang L. (2022). Techniques of double-pipe and double-acting
directional drilling
with air in broken and soft coal seams in underground coal mines. Coal Geol. Explor..

[107] Zhang J., Wang Y., Huang H., Wang C. (2018). Research on
directional drilling technology of air srew motor in soft coal seam. Coal Sci. Technol..

[108] Song Y. (2024). Soft coal drilling technology and application based on air directional
drilling. J. Phys..

[109] Xu, B. ; Wang, L. ; Wang, Y. ; Huang, H. ; Hou, H. ; Yao, Y. ; Jia, M. The broken soft seam flexibility internal control spin orientation drilling system of underground coal mine and drilling method; CN 107,605,402 B, 2017.

[110] Fang J., Li Q., Xu C., Liu J. (2018). Construction
technology and development tendency of gas drainage borehole in soft
and outburst seam. Coal Sci. Technol..

[111] Sun X., Wang L., Fang Y., Sun R., Jin X., Hou H. (2013). Technology and equipment of screen
pipe protected borehole of gas
drainage in soft seam. Coal Sci. Technol..

[112] Gao Y. (2014). Gas drainage based on screen pipe
placing in borehole technology
without drilling rod lifting in soft seam. Coal
Sci. Technol..

[113] Liu Q., Tong B., Fang Y., Tu Q., Guo S. (2014). High efficient
gas drainage technology with fast full length screen pipe for borehole
protection in soft seam. Coal Sci. Technol..

[114] Wang Z., Yang X., Wang G., Gong H. (2023). Study on instability
characteristics of the directional borehole on the coal-seam roof:
a case study of the tingnan coal mine. Processes.

[115] Liu C., Zhou F., Yang K., Xiao X., Liu Y. (2014). Failure analysis
of borehole liners in soft coal seam for gas drainage. Eng. Failure Anal..

[116] Peng Y., Fu G., Sun X., Sun B., Wang J., Zhang W., Du Q. (2022). Optimization design
of screen pipes hole arrangement parameter based on collapse strength. Thin Walled Struct..

[117] Zhang J., Wang Y., Yao N., Jin X., Zhang J., Wang L., Chen B., Ma T. (2020). Analysis of
coal particles passing screen pipe and test of gas drainage effect
in underground coal mine. J. Chinacoal Soc..

[118] Cheng, Y. Research On Failure Mechanisms Of Gas Drainage Through Drilling In Coal Seam And Efficient Sealing Technology; Thesis, China University of Mining and Technology, 2014.

[119] Ling, W. Experimental Study And Application Of Foam Concrete For Gas Extraction Borehole In Soft Coal Seams; Thesis, Xi’an University of Science and Technology, 2020.

[120] Cheng R., Zhang C., Fan F., Duan C., Chen Z. (2023). Research on hole collapse monitoring technology of coal seam gas
extraction boreholes. Sustainability.

[121] Wang G., Wang X., Zhao J., Zhang F., Wei H., Bai J. (2025). Research on inversion
method for stress characteristics
of roadway surrounding rock based on real-time weight measurement
of drill cuttings. Simulat. Modell. Pract. Theory.

[122] Zong C., Liu J., Liu Z., Liu Y., Wang C., Gao H., Duan J., Zhou J. (2023). A fast forward
algorithm of azimuthal gamma imaging logging while drilling. Appl. Radiat. Isot..

[123] Wang J., Wang C., Huang J., Han Z., Zeng W., Wang Y. (2022). In situ stress measurement method
of deep borehole based on multi-array ultrasonic scanning technology. Front. Earth Sci..

[124] Zou X., Song H. (2021). The fast formation of high-precision panoramic image
for the processing of borehole camera video of deep rock mass structures. Bull. Eng. Geol. Environ..

[125] Su X., Liu X., Ma B., Pei G., Feng W. (2014). Repairing
and enhancing permeability technology and equipment of gas drainage
borehole. Coal Sci. Technol..

